# Transgenic Cross-Referencing of Inhibitory and Excitatory Interneuron Populations to Dissect Neuronal Heterogeneity in the Dorsal Horn

**DOI:** 10.3389/fnmol.2020.00032

**Published:** 2020-04-17

**Authors:** Tyler J. Browne, Mark A. Gradwell, Jacqueline A. Iredale, Jessica F. Madden, Robert J. Callister, David I. Hughes, Christopher V. Dayas, Brett A. Graham

**Affiliations:** ^1^School of Biomedical Sciences & Pharmacy, Faculty of Health, University of Newcastle, New Lambton Heights, NSW, Australia; ^2^Hunter Medical Research Institute (HMRI), Callaghan, NSW, Australia; ^3^Institute of Neuroscience Psychology, College of Medical, Veterinary & Life Sciences, University of Glasgow, Glasgow, United Kingdom

**Keywords:** pain, spinal cord, neuron classification, patch clamp, excitability, neuron morphology

## Abstract

The superficial dorsal horn (SDH, LI-II) of the spinal cord receives and processes multimodal sensory information from skin, muscle, joints, and viscera then relay it to the brain. Neurons within the SDH fall into two broad categories, projection neurons and interneurons. The later can be further subdivided into excitatory and inhibitory types. Traditionally, interneurons within the SDH have been divided into overlapping groups according to their neurochemical, morphological and electrophysiological properties. Recent clustering analyses, based on molecular transcript profiles of cells and nuclei, have predicted many more functional groups of interneurons than expected using traditional approaches. In this study, we used electrophysiological and morphological data obtained from genetically-identified excitatory (vGLUT2) and inhibitory (vGAT) interneurons in transgenic mice to cluster cells into groups sharing common characteristics and subsequently determined how many clusters can be assigned by combinations of these properties. Consistent with previous reports, we show differences exist between excitatory and inhibitory interneurons in terms of their excitability, nature of the ongoing excitatory drive, action potential (AP) properties, sub-threshold current kinetics, and morphology. The resulting clusters based on statistical and unbiased assortment of these data fell well short of the numbers of molecularly predicted clusters. There was no clear characteristic that in isolation defined a population, rather multiple variables were needed to predict cluster membership. Importantly though, our analysis highlighted the appropriateness of using transgenic lines as tools to functionally subdivide both excitatory and inhibitory interneuron populations.

## Introduction

The superficial dorsal horn (SDH) of the spinal cord receives and processes primary afferent input conveying noxious, thermal, tactile, and pruritic sensory signals from the body destined for the brain. A key feature of the SDH, which includes laminae I and II, is the considerable heterogeneity within the local interneurons that make up >95% of the neurons in this region (Todd, 2010; Braz et al., 2014; Ross et al., 2014). In contrast, projection neurons, which are responsible for relaying sensory signals out of the SDH to the brain are scarce, accounting for <5% of neurons in this region (Polgár et al., 2010; Cameron et al., 2015). With this skew in the proportions of output to modulatory neurons, it is widely accepted that LI-II interneurons play a crucial role in processing sensory information before it is transmitted to the brain (Todd, 2010; Peirs and Seal, 2016).

Recent models of spinal sensory processing have provided a framework for understanding how interneurons contribute to spinal processing mechanisms. Specifically, they have highlighted the important interplay between inhibitory gating mechanisms (Bourane et al., 2015; Foster et al., 2015; Petitjean et al., 2015; François et al., 2017; Sun et al., 2017; Boyle et al., 2019) and polysynaptic excitatory pathways (Torsney and MacDermott, 2006; Takazawa and MacDermott, 2010; Peirs et al., 2015; Takazawa et al., 2017) for normal sensory experience. As these models have developed, so too has the need to differentiate subpopulations of interneurons with unique features and connections that can fulfill specific roles in spinal sensory circuits. For example, intersectional transgenic ablation and optogenetic activation studies have implicated somatostatin-expressing dorsal horn neurons in mechanical pain (Duan et al., 2014; François et al., 2017), whereas dynorphin (Dyn)-expressing neurons suppress this action (Duan et al., 2014). Chemogenetic manipulation of transiently expressing VGLUT3 neurons has shown these cells receive touch-related sensory inputs from myelinated afferents and relay them through a circuit implicated in generating mechanical allodynia (Peirs et al., 2015). Genetic ablation and chemogenetic activation of parvalbumin (PV) expressing inhibitory interneurons have also implicated the PV population in mechanical allodynia (Petitjean et al., 2015). Similar observations have also been reported using tetanus toxin-based silencing of the PV interneurons with detailed optogenetic circuit mapping confirming these cells are a source of presynaptic inhibition on myelinated afferents, as well as mediating postsynaptic inhibition to excitatory interneurons that relay touch-related signals into lamina I (Boyle et al., 2019).

Alternatively, experiments ablating interneurons expressing the gastrin-releasing peptide (GRP) receptor have shown this population to be integral for spinal itch transmission (Sun et al., 2017). Subsequent optogenetic activation studies of GRP-expressing neurons in the same region highlighted the action of GRP in volume transmission and its ability to also produce itch responses in transgenic mice (Pagani et al., 2019). Finally, using a combination of chemogenetics and targeted ablation approaches, somatostatin-mediated activation of Dyn expressing interneurons has also been implicated in spinal itch signaling (Huang et al., 2018).

While far from exhaustive, the above snapshot of recent work on the involvement of interneuron types in pain and itch signaling highlights the value of these new approaches for understanding spinal sensory processing circuits. However, it also demonstrates the piecemeal and often overlapping nature of these studies. An improved understanding of spinal sensory processing will require these discrete microcircuits and connections to be examined in a way that better accounts for the well-known neuron heterogeneity that exists in the SDH.

Advances in single-cell and single nucleus sequencing techniques have provided a new approach for differentiating distinct neuron subpopulations (Zeisel et al., 2015). These approaches have the advantage of simultaneously comparing and then subdividing the entire dorsal horn population. This provides a complete taxonomy/classification for nonoverlapping neuronal subpopulations based on their molecular profile. Using the single-cell approach, 30 different dorsal-horn neuron types have been identified, 15 excitatory and 15 inhibitory (Haring et al., 2018). A similar exercise using single nucleus expression data identified 16 excitatory and nine inhibitory neuron classes (Sathyamurthy et al., 2018). These results far exceed the number of cell types predicted by the existing literature, which subdivides the dorsal horn population using morphological, electrophysiological or neurochemical properties (Grudt and Perl, 2002; Yasaka et al., 2010; Gutierrez-Mecinas et al., 2016, 2019; Boyle et al., 2017).

One result of these two broad approaches for classifying neuron types is that as more and more neurochemically defined interneurons are identified and classified, they will often span several molecularly defined classes. For example, somatostatin expression occurs in 10 of the 15 excitatory molecular subclasses, while PV and Dyn are expressed in 3 of the 15 inhibitory molecular subclasses. A simple interpretation of this relationship is that either smaller subpopulations exist within the neuron types that have been studied functionally. Alternatively, multiple distinct molecular cell classes of neurons show phenotypic convergence and assume the same role in spinal sensory processing circuits (Zeisel et al., 2015; Harris et al., 2018). The possibility remains that phenotypic divergence also exists, where cells in a single molecular class can assume distinct roles within sensory circuits. Thus, reconciling the relationship between molecular taxonomies and functional approaches to neuronal characterization will be important for our future understanding of heterogeneity in the SDH.

To address this challenge, we have adopted a population-level approach to functional characterization. We generated mice where the fluorescent reporter protein, TdTomato, is expressed in neurons containing the vesicular γ-aminobutyric acid transporter (vGAT) or the vesicular glutamate transporter-2 (vGLUT2). This enabled us to study inhibitory and excitatory dorsal horn neurons, respectively. Several studies have also used reporter proteins (eg GFP/TdTomato) expressed in specific cell types to study excitatory and inhibitory populations (Hantman et al., 2004; Heinke et al., 2004; Zeilhofer et al., 2005; Hughes et al., 2012; Duan et al., 2014; Punnakkal et al., 2014; Smith et al., 2015; Dickie et al., 2019). Electrophysiological and morphological data from the labeled populations in our experiments, as well as adjoining unlabeled cells, were compared to resolve features unique or shared within each cell type and group. Our results reinforce and highlight a number of features that differ between cell classes, including action potential (AP) spiking, subthreshold current activation and excitatory synaptic drive. Likewise, some key morphological features also segregate cell classes, however, no electrophysiological or morphological property was uniquely expressed in one population, cautioning against using these features to predict cell type. Finally, in an attempt to account for the large numbers of interneuron populations identified using molecular profiles we used both electrophysiological and morphological data to form hierarchical clusters. Similar exercises in other CNS regions including the cortex (Cauli et al., 2000; Gouwens et al., 2019) and hippocampus (Graves et al., 2012) have successfully resolved functionally discrete subpopulations. In the dorsal horn, we show that electrophysiological properties better differentiate cell classes than morphological characteristics. This analysis lends support to the existence of functionally distinct subpopulations of excitatory and inhibitory dorsal horn interneurons, though far less than proposed using molecular expression data.

## Materials and Methods

Mice for experiments were generated by crossing either a VGAT−Cre line [B6J.129S6(FVB)-Slc32a1^tm2(cre)Lowl^/MwarJ, Jackson Laboratories, Bar Harbor, ME, USA, #028862] or, a VGLUT2−Cre line [B6J.129S6(FVB)-Slc32a1^tm2(cre)Lowl^/MwarJ, Jackson Laboratories, Bar Harbor, ME, USA, #028863] with a mouse reporter line to express a Cre-dependent~Td tomato [B6.Cg-Gt(ROSA)26Sortm14(CAG-TdTomato)Hze/J, Jackson Laboratories, Bar Harbor, ME, USA, #007914]. This generated mice where TdTomato is expressed in either vGAT (inhibitory) or vGLUT2 (excitatory) positive neurons. Both Cre-lines have previously been used to manipulate these populations in the dorsal horn using chemogenetics (Koga et al., 2017; Wang et al., 2018), validating their utility in the dorsal horn and highlighting the need for a detailed electrophysiological and morphological characterization. All experimental procedures were performed in accordance with the University of Newcastle’s animal care and ethics committee. Animals of both sexes (aged 4–7 months) were used for electrophysiology and subsequent morphological analyses. This age range ensured data was collected from mature animals at a stage where electrophysiological properties are thought to be stable. This is on the basis of work from our group showing significant alterations in the electrophysiological properties of DH neurons that occur very early in postnatal development—either side of a critical developmental period from P6 to P10 (Walsh et al., 2009). In other work, we have shown there are subtle but significant changes in excitability, excitatory drive, and GABA signaling between young adulthood (3–4 months) and advanced age (28–32 months; Mayhew et al., 2019). Taken together, this supports the view our comparisons are unlikely to be influenced by animal age.

### Tissue Preparation for Immunocytochemistry

To assess the validity of TdTom expression in vGAT expressing neurons as a marker for inhibitory interneurons in the dorsal horn, we assessed co-expression of an accepted developmental marker for all inhibitory interneurons, Pax2, in this tissue (Foster et al., 2015; Boyle et al., 2017; Larsson, 2017). Pax2 expression was also assessed in vGLUT2 animals to validate our capacity to identify excitatory interneurons—the expectation being no overlap between TdTom and Pax2 expression in the vGLUT2 tissue. Briefly, animals (*n* = 3, vGAT:TdTom and vGLUT2:TdTom) were overdosed with sodium pentobarbitone (30 mg/kg i.p) and perfused transcardially with 0.9% sodium chloride solution followed by 4% fresh depolymerized formaldehyde in 0.1 M phosphate buffer (pH 7.4). Spinal cords were dissected out and post-fixed in the same fixative for 2 h. Transverse sections (30 μm thick) were cut from the lumbar enlargement (L3–L5) on a cryostat (CM1900 Leica, Wetzlar, Germany) at −20°C. Sections were then incubated in primary antibody, anti-Pax2 (Invitrogen—Thermo Fisher, Waltham, MA, USA, #71-6000), reacted with a secondary antibody conjugated to Cy5 (Jackson Immunoresearch Laboratories, West Grove, PA, USA), before mounting on slides in glycerol. Image stacks (>10 optical sections) were captured using laser-scanning confocal microscopy on a Leica TCS SP8 microscope (equipped with 405 nm diode, argon multiline, solid-state, and HeNe lasers) using a 10×/0.4 apochromat air interface objective (field of view: 1,117 × 1,117 μm, *z* = 2 μm, pinhole 1 AU), or 40×/1.3 apochromat oil immersion objectives (field of view: 281 × 281 μm, *z* = 1 μm, pinhole: 1 AU) to capture “whole of dorsal horn” and higher resolution images, respectively.

### Analysis of Confocal Image Stacks

Images were analyzed offline using the open-source image processing software Fiji (including the cell counting plugin; Schindelin et al., 2012). Three separate areas (100 μm × 100 μm square superimposed) were sampled across the dorsal horn including a medial area, bordering on the dorsal columns; lateral area, bordering on the lateral spinal nucleus and a middle position (between medial and lateral). Cells were counted in image stacks and only marked in the analysis only if they could be visualized in four or more adjacent optical sections. Each channel representing a neurochemical marker (vGAT/vGLUT2~Tdtomato, or Pax2~Cy5) was visualized separately. All positive profiles on each channel were marked without observing the other channel, before merging the fields and assessing the overlap between Tdtomato and Pax2, or lack thereof. Maximum intensity Z-projections of images were generated to summarize these distributions.

### Spinal Cord Slice Preparation

Spinal cord slices for patch-clamp electrophysiology were prepared using previously reported methods (Smith et al., 2015). In brief, animals were anesthetized with ketamine (100 mg/kg i.p) and decapitated. The ventral surface of the vertebral column was exposed, and the spinal cord rapidly dissected out (within 8–10 min) in ice-cold sucrose substituted cerebrospinal fluid (ACSF) containing (in mM): 250 sucrose, 25 NaHCO_3_, 10 glucose, 2.5 KCl, 1 NaH_2_PO_4_, 1 MgCl_2_ and 2.5 CaCl_2_. All recordings were undertaken in parasagittal slices (L1-L5, 200 μm thick), prepared using a vibrating microtome (Campden Instruments 7000 smz, Loughborough, UK). Slices were transferred to an air interface incubation chamber containing oxygenated ACSF (118 mM NaCl substituted for sucrose) and allowed to equilibrate at room temperature (~23°) for at least 1 h prior to recording.

### Patch-Clamp Electrophysiology

**Figure 1:**
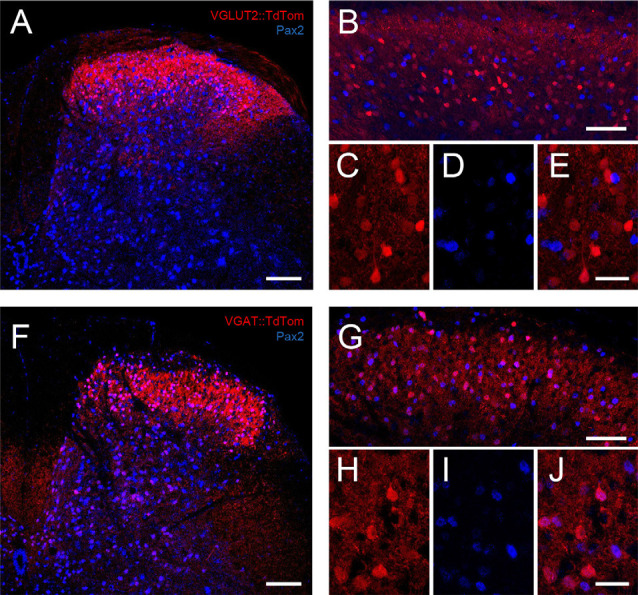
TdTomato expression in VGLUT2:Td and VGAT:Td dorsal horn neurons. **(A)** Low magnification image is a maximum intensity z-projection showing the distribution of TdTom expression (red) in the dorsal horn of a spinal cord section (medial to left) from a VGLUT2:Td mouse merged with Pax2 immunolabeling (blue), which identifies inhibitory neurons. **(B)** A higher magnification image is a maximum intensity z-projection also showing the distribution of VGLUT2:Td and Pax2 expression within the superficial dorsal horn (SDH). Note, the lack of overlap of red and blue cell profiles. **(C–E)** Expanded images (from **B**) show VGLUT2:Td, Pax2, and both merged, highlighting the lack of overlap and confirming VGLUT2:Td expression is limited to excitatory (Pax2−) profiles. **(F)** Low magnification image is a maximum intensity z-projection showing the distribution of TdTom expression (red) across the dorsal horn of a spinal cord section from a VGAT:Td mouse merged with Pax2 immunolabeling (blue) to identify inhibitory neurons. **(G)** Higher magnification image is a maximum intensity z-projection also showing the distribution of VGAT:Td and Pax2 expression within the SDH. Note, the substantial overlap. **(H–J)** Expanded images (from **G**) show VGAT:Td, Pax2, and both merged. The overlap of fluorescent profiles confirms VGAT:Td expression is commonly found in inhibitory (Pax2+) profiles (pink; Scale bars: **A,B,F,G**: 100 μm; **C–E,H–J**: 25 μm).

Following incubation, slices were transferred to a recording chamber and continuously superfused with Carbanox-bubbled ACSF (95% O_2_, 5% CO_2_) to achieve a final pH of 7.3–7.4. All recordings were made at room temperature. Neurons were visualized using a 40× objective and near-IR differential interference contrast optics. To identify vGAT and vGLUT2 positive neurons that expressed red fluorescent protein (RFP; TdTomato), slices were viewed under fluorescence using a TRITC filter set. vGAT and vGLUT2 neurons within substantia gelatinosa (LI-II) were targeted for recordings and were easily identified by their clear somatic fluorescence (Figure 1). In addition, some recordings also selectively targeted unlabeled (TdTom negative) neurons in tissue from vGAT/vGLUT2:TdTomato animals. All recordings used patch pipettes (4–8 MΩ) filled with a potassium gluconate based internal containing (in mM): 135 C_6_H_11_KO_7_, 6 NaCl, 2 MgCl_2_, 10 HEPES, 0.1 EGTA, 2 MgATP, 0.3 NaGTP, pH 7.3 (with KOH), to record excitatory synaptic input and AP discharge (series resistance <40 MΩ). Neurobiotin (0.2%) was included in the internal solution for studying *post hoc* cell morphology. All data were acquired using a Multiclamp 700B amplifier (Molecular Devices, Sunnyvale, CA, USA), digitized online (sampled at 10 kHz, filtered at 5 kHz) using an ITC-18 computer interface (Instrutech, Long Island, NY, USA) and stored on the computer. This data capture and subsequent analysis used Axograph X software (Molecular Devices, Sunnyvale, CA, USA).

AP discharge patterns were assessed in the current clamp from a membrane potential of −60 mV by delivering a series of depolarizing current steps (1 s duration, 20 pA increments), with rheobase defined as the first current step that induced AP discharge. AP discharge patterns were classified using previously described criteria (Graham et al., 2004, 2007, Graham et al., 2008). Briefly, delayed firing (DF) neurons exhibited a clear, ramped interval between current injection and the onset of the first AP; tonic firing (TF) neurons exhibited continuous repetitive AP discharge for the duration of current injection; initial bursting (IB) neurons were characterized by a burst of AP discharge at the onset of current injection, and single spiking (SS) neurons only fired a single AP at the beginning of the current step. In addition, we observed one rarely described discharge profile that we termed a rapid burst (RB) response. In these recordings, a rapid burst of AP discharge occurred during a pronounced depolarizing current hump at the rheobase step, which is consistent with the activation of low threshold T-type calcium channels. While the resulting spiking in this population could be classified as IB, their unique underlying features clearly differentiated these responses and warranted classification as a distinct group.

The subthreshold currents underlying AP discharge were identified in a voltage-clamp protocol. This protocol consisted of an initial hyperpolarization from a holding potential of −70 mV to −100 mV (1 s duration) followed by a depolarizing step to −40 mV (200 ms duration) and applied P/N leak subtraction for analysis (within Axograph software). Four major ionic currents previously described in dorsal horn neurons could be identified in responses, including the outward potassium currents (I_A_) and the inward currents; T-type calcium and the non-specific cationic current, I_h_. Input resistance and series resistance were monitored throughout all recordings and data were excluded if either value changed by more than 10%. No correction was made for liquid junction potentials.

### Patch-Clamp Data Analysis

All electrophysiology data were analyzed offline using Axograph X software. Input resistance was calculated from the averaged current response (30 trials) generated in each cell during a 5 mV hyperpolarizing step. Spontaneous excitatory postsynaptic currents (sEPSCs) were detected using a sliding template method (a semi-automated procedure in the Axograph package). sEPSCs frequency was determined from at least 30 s of continuous recording at a membrane potential of −70 mV. Peak amplitude, rise time (10–90% of peak), and decay time constant (fitted over 10–90% of the decay phase) were measured from the averaged sEPSC obtained for each neuron. The excitatory drive was calculated as the area under the curve of the averaged sEPSC multiplied by sEPSC frequency. Resting membrane potential (RMP) was taken as the average of 30 s of current clamp recording immediately after entering the whole-cell mode (input current = 0 pA). Individual AP properties for each neuron were assessed from the first spike generated at rheobase current. APs were captured using a derivative threshold method with AP threshold defined as the inflection point during spike initiation (dV/dt ≥15 V/s). The difference between the AP threshold and its maximum positive peak was defined as the AP peak. AP half-width was measured at 50% of AP peak, and AP afterhyperpolarization (AHP) amplitude was taken as the difference between the AP threshold and the maximum negative peak following the AP. AP discharge pattern properties were all assessed in the response to multiple steps with the response exhibited at two-step injections above rheobase (Rh40) used to measure key characteristics. AP delay was the time between step onset and the first AP discharged during the response. The first interspike interval (1st AP ISI) was the time between the onset of the first and second APs in the response. AP duration was the time between the first and last AP in the step response. AP number (AP n) was the number of spikes discharged in the entire response, and spiking AP adaptation was the ratio of the first interspike interval divided by the last interspike interval. For subthreshold currents, I_A_ was identified by an outward positive peak in the depolarizing step of the subthreshold current protocol (−100 mV to −40 mV) whereas I_Ca_ was identified as an inward current during this step. Peak current was measured (positive or negative) and used to designate cells as exhibiting dominant I_A_ or I_Ca_ (or no current) responses. This approach cannot exclude the possibility that some cells expressed both I_A_ and I_Ca_ currents, though even if occurring in combination our protocol yields the current likely to have the greatest functional relevance for spiking by predominating. I_h_ was characterized by a sag in the hyperpolarizing step (−70 mV to −100 mV). Peak I_h_ current was quantified as the difference between the immediate current response to this step subtracted from the maximum steady-state current at the end of this step.

### Anatomical Characterization of vGAT and vGLUT2~TdTomato Neurons

As noted above, Neurobiotin (0.2%) was included in the recording pipette for morphological analysis of recorded neurons. Slices containing filled cells were incubated in Streptavidin-Alexafluor 488 secondary antibody (1:50, 2 h, Thermofisher Scientific, #S11223). They were mounted on slides with glycerol and imaged on a Leica TCS SP8 scanning confocal microscope using a 25 × 0.95 fluotar-water immersion objective (working distance 2.4 mm, *z* = 1 μm, the field of view 445 μm × 445 μm). The dendritic architecture was analyzed for all cell recoveries deemed adequate to assess the rostrocaudal and dorsoventral extent occupied by processes. Specifically, as all recordings were undertaken in sagittal slices, the dorsoventral and rostrocaudal axes of each image were first oriented. Only cells with a clearly differentiated filled soma and processes, not overlapping with other filled cells in the same slice were analyzed. Three measurements were taken for each cell of these images: (1) the rostrocaudal extent, which was the distance between the most rostral and caudal labeled processes in the rostrocaudal plane; (2) the dorsal extent, which was the distance between the center of the cells soma and the dorsal-most labeled process in the dorsoventral plane; and (3) the ventral extent, which was the distance between the center of the cells soma and the ventral-most labeled process in the dorsoventral plane. A series of measures were also derived from this data, with dorsal and ventral measures added to yield dorsoventral extent, rostrocaudal extent was divided by the dorsoventral extent to provide a rostrocaudal/dorsoventral (RC:DV) ratio, and dorsal extent was subtracted from the ventral extent to yield a measure of dorsoventral bias. These measures have previously been shown to efficiently distinguish neuronal morphology in the dorsal horn (Alba-Delgado et al., 2015). Processes with axonal characteristics (small, non-tapering diameter) were also occasionally observed within dendritic profiles and beyond, however, when present their distribution was not assessed in this study.

### Clustering Analysis: Electrophysiological and Morphological Parameters

Clustering analyses were completed using Orange (v3.2) data analysis software (Demšar et al., 2013). Electrophysiological, morphological and combined (i.e., electrophysiology and morphology) datasets were imported into the Orange data mining toolbox. Euclidean distance was calculated for these values and hierarchical cluster analyses were completed using Ward’s minimum variance method. Given the potential for variables with larger scales to overly influence clustering, Euclidean distance calculations and clustering was also repeated with scale of all variables normalized (0–1). Clustering was identical between raw and normalized data and thus outputs from the least processed (raw) data are presented. Dendrograms were generated for cluster analysis outputs and heat maps were obtained for each property included in these analyses—with the color range scaled to maximum (brown-red) and minimum (white-beige) values. A parsimonious cluster number was determined for each analysis using the K-means elbow method with the sum of squared errors (SSE) calculated for *n* = 2 to 15 clusters. Under this approach, the number of clusters was assigned for each hierarchical analysis (electrophysiology, morphology, electrophysiology and morphology) by determining where additional clusters no longer yielded an appreciable improvement to the SSE.

### Statistical Analysis

All data are presented as mean ± standard deviation (SD) unless otherwise stated. One-way ANOVAs were performed with a student Newman–Keuls *post hoc* test to compare electrophysiological and morphological properties between neuron classes (identified inhibitory, identified excitatory, putative inhibitory, and putative excitatory). Chi-squared tests compared the distribution of AP discharge patterns and subthreshold current expression between neuron classes.

## Results

The following results include data from 198 dorsal horn neurons recorded in the SDH (laminae I-II) of spinal cord slices prepared from VGAT:TdTomato (vGAT:Td, *n* = 6) and VGLUT2:TdTomato mice (vGLUT2:Td, *n* = 3). In each transgenic mouse line, recordings were targeted to labeled TdTomato positive neurons (vGAT+, vGLUT2+) and unlabeled TdTomato negative neurons (VGAT−, VGLUT2−). Thus, four-cell groups are differentiated and reported on: (1) targeted inhibitory interneurons (VGAT+, *n* = 76); (2) putative excitatory interneurons (VGAT−, *n* = 57); (3) targeted excitatory interneurons (VGLUT2+, *n* = 40); and (4) putative inhibitory interneurons (VGLUT2−, *n* = 25). It follows that VGAT+ and VGLUT2− samples should come from the same overall (inhibitory) population, and likewise, the VGLUT2+ and VGAT− (excitatory) samples should overlap substantially.

### TdTomato Labeling in VGAT:Td and VGLUT2:Td Mice

To assess our capacity to reliably identify inhibitory and excitatory interneurons in VGAT:Td and VGLUT2:Td mice, we first undertook immunolabeling experiments. TdTomato-expression (TdTom) in the lumbar spinal cord sections from each genotype was compared to Pax2 immunolabeling (five sections per animal from three mice per genotype), which provides a reliable marker of inhibitory neuron phenotype in the dorsal horn (Figure 1). Cell counts were undertaken in standardized “regions of interest” (100 μm × 100 μm) superimposed on the medial, middle, and lateral SDH to assess the likelihood that TdTom+ cell distribution was concentrated in a particular region. There was no difference in the number of TdTom+ cells across the mediolateral axis in VGAT:Td sections (mean ± SD cells per section = 17.9 ± 4.6 vs. 21.3 ± 4.9 vs. 20.3 ± 4.5, *p* = 0.144; lateral vs. middle vs. medial, respectively). In contrast, TdTom+ cell counts were lower in the medial dorsal horn compared to the middle region counts in VGLUT2:Td sections (20.5 ± 7.7 vs. 23.7 ± 3.4 vs. 18.1 ± 6.1 cells per section, *p* = 0.047; lateral vs. middle vs. medial, respectively). Despite this minor regional difference for the VGLUT2:Td tissue, the number of TdTom+ cells per section was similar between VGAT:Td and VGLUT2:Td animals (59.5 ± 10.2 vs. 62.3 ± 10.8 cells per section, *p* = 0.471). Likewise, the number of Pax2+ cells was similar between VGAT:Td and VGLUT:Td animals (64.2 ± 11.3 vs. 60.3 ± 8.5 cells per section, *p* = 0.297). This analysis rules out inconsistent labeling as a cause for any subsequent difference in electrophysiological or morphological comparisons. In contrast, differences in the number of TdTom+ cells that exhibited overlapping Pax2+ immunolabeling in VGAT:Td vs. VGLUT2:Td animals were dramatic. For the VGAT:Td animal: 93.9% of TdTom+ cells were Pax2+, 87.3% of Pax2+ profiles were TdTom+, and 6.1% of TdTom+ did not express Pax2. This shows cells labeled in the vGAT:Td line are mostly inhibitory, some inhibitory interneurons are undetected, and importantly the chance of falsely identifying inhibitory interneurons is low.

As an excitatory cell marker was not readily available for this study a similar analysis to that above was not possible for the VGLUT2:Td animals. We could, however, use an exclusion approach to assess the capacity of TdTom+ expression to reliably identify excitatory interneurons by showing 91.6% of TdTom+ cells were Pax2−, and only 8.3% of TdTom+ cells were Pax2+. These data indicate that almost all vGLUT2 profiles were not inhibitory and thus the chance of falsely identifying an excitatory interneuron was low. The fraction of excitatory interneurons captured by the VGLUT2:Td animal could not be assessed without an excitatory cell marker. This value can, however, be estimated by comparison with the VGAT:Td animal using the well-established ratio of excitatory to inhibitory interneurons in the dorsal horn (~75%:25%; Polgár et al., 2013; Gutierrez-Mecinas et al., 2019). As the VGAT:Td animals captured most inhibitory interneurons, but similar cell numbers are labeled in VGAT:Td and VGLUT2:Td animals, we estimate the VGLUT2:Td animal captures ~30% of the excitatory interneurons in the region. Thus, a population of excitatory interneurons appears to remain unidentifiable (TdTom−) in this tissue.

### Distinct Electrophysiological Properties in VGAT:Td and VGLUT2:Td Populations

A comparison of the electrical properties across excitatory and inhibitory interneurons revealed some differences between these populations (Table 1). First, the input resistance of the inhibitory VGAT+ group was lower than the excitatory VGLUT2+ group (*p* < 0.001). This difference was also conserved between inhibitory VGAT+ cells and putative excitatory VGAT− cells (*p* < 0.001). Likewise, input resistance was lower in putative inhibitory (VGLUT2−) cells vs. excitatory VGLUT2+ cells (*p* = 0.05), but not in the putative excitatory VGAT− population. Differences in RMP were less prominent, with only targeted inhibitory VGAT+ cells exhibiting more depolarized membrane potentials than excitatory VGLUT2+ cells (*p* = 0.04). Finally, the rheobase current required to initiate AP discharge did not differ across the four neuron groups.

**Table 1:** Passive membrane properties.

Cell type	Input resistance (MΩ)	RMP (mV)	Rheobase (pA)
VGAT+ (*targeted inhibitory*, *n* = 76)	170 ± 99* (VGAT−, VGLUT2+)	−55.5 ± 10.1* (VGLUT2+)	51.9 ± 22.4
VGAT− (*putative excitatory*, *n* = 57)	270 ± 133* (VGAT+)	−59.4 ± 12.8	48.1 ± 26.3
VGLUT2+ (*targeted excitatory*, *n* = 40)	303 ± 178* (VGAT+, VGLUT2−)	−61.5 ± 12.8	48.2 ± 26.6
VGLUT2− (*putative inhibitory*, *n* = 25)	214 ± 128* (VGLUT2+)	−59.3 ± 11.7	47.0 ± 23.8

Mean ± SD. Significant differences between groups denoted by *(bracketed below, *p* < 0.05).

Despite a similar overall rheobase current, the properties of rheobase APs did differ (Table 2). Specifically, the AP threshold was more hyperpolarized in the targeted and putative inhibitory populations (VGAT+ and VGLUT2−) compared to the targeted and putative excitatory populations (VGLUT2+ and VGAT−; *p* < 0.001, Student Newman–Keuls *post hoc* test). The amplitude of APs was larger for the inhibitory cell groups compared to the excitatory populations (VGAT+ and VGLUT2− vs. VGLUT2+ and VGAT−, *p* < 0.001), and the time course (rise time and half-width) was faster in the inhibitory vs. excitatory subsets (VGAT+ and VGLUT2− vs. VGLUT2+ and VGAT−, *p* < 0.001). Finally, AHP amplitude was larger in VGLUT2− vs. VGLUT2+ neurons (*p* = 0.02). Thus, inhibitory dorsal horn populations exhibited AP spikes that had larger amplitudes and faster time courses than their excitatory counterparts.

**Table 2:** Action potential properties.

Cell type	AP threshold (mV)	AP peak (mV)	AP rise time (ms)	AP half-width (ms)	AHP peak (mV)
VGAT+ (*targeted inhibitory*, *n* = 74)	−33.2 ± 3.7* (VGAT−, VGLUT2+)	63.7 ± 11.7* (VGAT−, VGLUT2+)	0.86 ± 0.23 (VGAT−, VGLUT2+)	3.18 ± 0.78 (VGAT−, VGLUT2+)	−20.5 ± 7.3
VGAT− (*putative excitatory*, *n* = 51)	−27.2 ± 6.0* (VGAT+, VGLUT2−)	52.8 ± 11.3 (VGAT+, VGLUT2−)	1.11 ± 0.40 (VGAT+, VGLUT2−)	3.90 ± 1.30 (VGAT+, VGLUT2−)	−20.9 ± 6.5
VGLUT2+ (*targeted excitatory*, *n* = 39)	−27.8 ± 6.0* (VGAT+, VGLUT2−)	49.9 ± 11.4 (VGAT+, VGLUT2−)	1.22 ± 0.48 (VGAT+, VGLUT2−)	3.99 ± 1.09 (VGAT+, VGLUT2−)	−18.1 ± 6.5 (VGLUT2−)
VGLUT2− (*putative inhibitory*, *n* = 23)	−30.2 ± 5.6* (VGAT−, VGLUT2+)	58.7 ± 9.7 (VGAT−, VGLUT2+)	0.80 ± 0.20 (VGAT−, VGLUT2+)	3.06 ± 0.87 (VGAT−, VGLUT2+)	−23.9 ± 5.8 (VGLUT2+)

Mean ± SD. Significant differences between groups denoted by *(bracketed below, *p* < 0.05).

**Figure 2:**
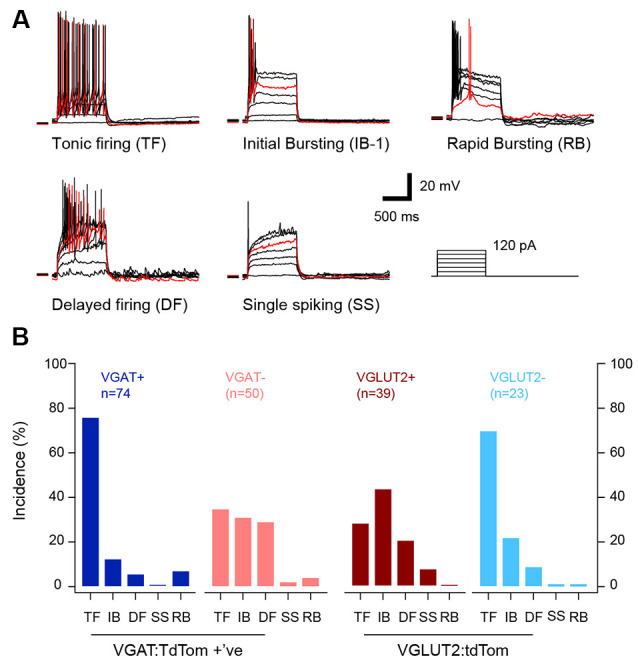
Distinct action potential (AP) discharge patterns in excitatory and inhibitory populations. **(A)** Overlaid traces show representative examples of the characteristic patterns of AP discharge observed during increasing levels of current step injection (20 pA steps, 1 s duration). The red trace shows an exemplary response for each AP discharge pattern. Tonic firing (TF) responses included repetitive AP discharge throughout the depolarizing current step injection; initial bursting (IB) responses exhibited AP discharge that was restricted to the depolarization onset; Rapid Bursting responses were similar to IB, but spike onset coincided with a depolarizing hump that was clearly visible in the rheobase current response; Delayed firing (DF) responses exhibited AP discharge only after a delay from the depolarizing step onset; and Single Spiking responses only discharged a single AP at depolarization onset, regardless of depolarizing step amplitude. **(B)** Bar plots compare the incidence of each AP discharge patterns between cell types. Most (>70%) inhibitory interneurons (VGAT+ and VGLUT2−, blue and light blue bars) exhibited TF and the incidence of IB, DF and rapid bursting was low (<20%). In contrast, excitatory populations (VGLUT2+ and VGAT−, brown and red bars) exhibited a wider range of AP discharge properties with IB, DF and TF each accounting for >20% of the distribution.

We next assessed the features of AP discharge initiated by depolarizing current step injection (1 s), because the literature suggests certain discharge responses are features of inhibitory and excitatory interneurons (Figure 2A). Consistent with our previous work, discharge patterns could be distinguished by distinct characteristics of repetitive AP discharge (Table 3). For example, TF was characterized by low rheobase, a high number of APs, and long discharge durations. In contrast, AP discharge commenced significantly later following step onset in DF, responses were limited to one AP in Sigle Spiking, and Rapid bursting exhibited significant spike adaptation. A comparison of the relative distribution of discharge responses across the inhibitory and excitatory populations (Figure 2B) highlighted important differences (*p* < 0.001). The TF was the dominant response among inhibitory interneurons (~70% vs. 30%; VGAT+/VGLUT2− vs. VGLUT2+/VGAT−). In contrast, IB and DF responses were more frequent in excitatory interneurons (IB ~35% vs. 15%, DF ~25% vs. 7%; VGLUT2+/VGAT− vs. VGAT+/VGLUT2−). Finally, although rare, rapid burst discharge (Figure 2A) was observed in VGAT+ and VGAT− neurons (7% and 4%, respectively).

**Table 3:** Action potential discharge pattern characteristics.

	TF	IB	DF	SS	RB
Rheobase (pA)	43.57 ± 17.61 *DF, SS, RB	47.06 ± 25.05 *DF, SS	71.30 ± 25.46 *TF, IB	110.00 ± 14.14 *TF, IB	74.29 ± 29.92 *TF
AP spiking delay (ms)	40.51 ± 18.89 *DF	32.60 ± 14.61 *DF	193.73 ± 128.25 *TF, IB, SS, RB	8.65 ± 2.76 *DF	20.01 ± 13.13 *DF
1st AP ISI (ms)	54.72 ± 25.68	69.56 ± 160.43	88.46 ± 143.75	-	95.45 ± 202.67
APs (*n*)	12.98 ± 6.97 *IB, SS, RB	6.38 ± 3.63 *TF	9.30 ± 5.52	1.00 ± 0.00 *TF	5.14 ± 2.48 *TF
AP spiking duration (ms)	861.42 ± 128.04 *IB, DF, RB	365.24 ± 262.93 *TF, DF	605.11 ± 277.12 *TF, IB	-	372.56 ± 348.35 *TF
AP spiking adaptation	0.49 ± 0.20 *RB	0.55 ± 0.29 *RB	0.85 ± 0.41 *RB	-	2.60 ± 5.71 *TF, IB, DF

*p < 0.05.

**Figure 3:**
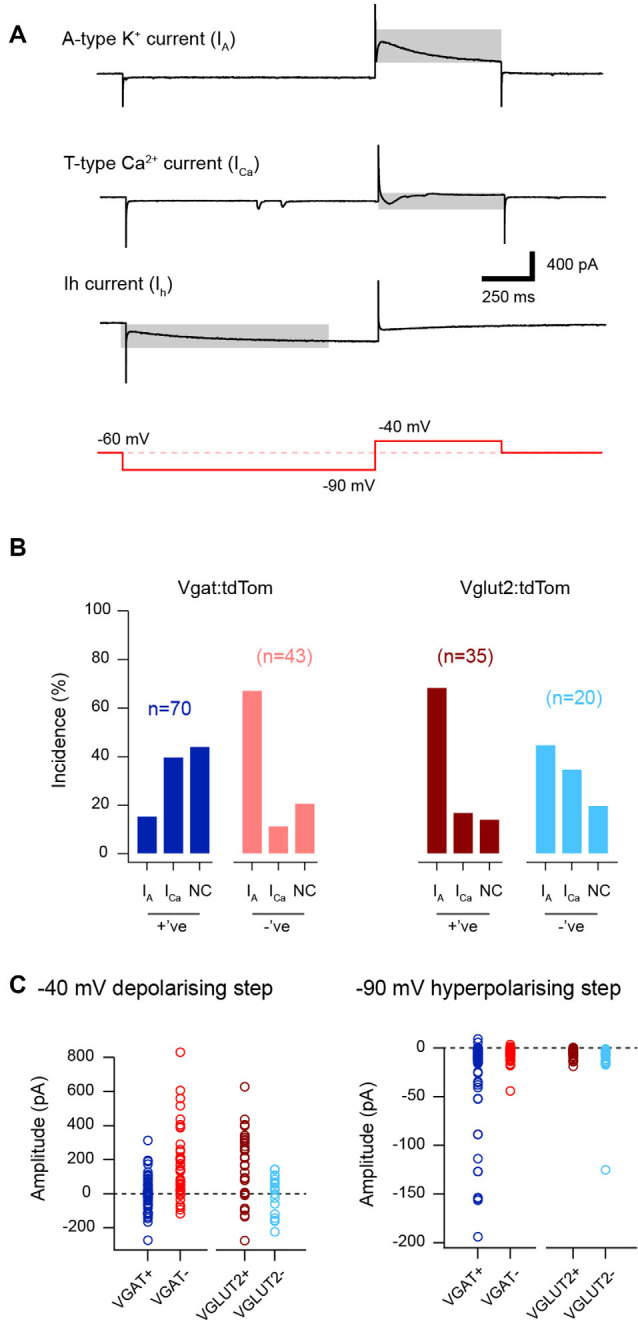
Subthreshold voltage-activated current expression in excitatory and inhibitory populations. **(A)** Traces show representative responses to a voltage step protocol (red lower trace). Three subthreshold voltage-activated currents are identified (highlighted by gray rectangle). These include outward A-type potassium currents (upper trace), inward low threshold T-type calcium currents (middle), and inward hyperpolarization-activated cation currents (I_h_, lower). **(B)** Plots show the relative incidence of voltage-activated currents taken from the response to a current step from −90 mV to −40 mV. A-type currents were frequently observed in recordings from excitatory populations (vGLUT2+ and VGAT−, brown and red bars) whereas T-type Ca-currents were more commonly expressed by inhibitory neurons (VGAT+ and VGLUT2−, blue and light blue bars). Note, between 15–40% of recordings in each population did not exhibit a subthreshold current response to this step and they are termed no current (NC) on the bar plots. **(C)** Plots showing the peak amplitude of the subthreshold current responses for the depolarizing −90 mV to −40 mV (left), and the hyperpolarizing −70 mV to −90 mV steps (right). The left plot shows the dominance of outward current values (i.e., above the dotted line showing zero pA) in the excitatory population, because of the dominance of A-type potassium currents in these cells. In contrast, the inhibitory populations (VGAT+ and VGLUT2−) exhibit smaller currents (i.e., near or below the dotted line), consistent with greater expression of inward T-type calcium currents. The right plot shows the amplitude of I_h_ in the four neuron types. Note, the large peak amplitude of I_h_, among the inhibitory populations (VGAT+ and VGLUT2−).

Consistent with the above distribution of AP discharge patterns, the subthreshold voltage-activated currents that shape AP discharge was also differentially expressed across the cell groupings (Figure 3). Specifically, voltage steps from −90 mV to −40 mV unmasked two opposing rapidly activating transient voltage-activated currents in LI-II neurons, either an outward A-type potassium current or an inward T-type calcium current (Figure 3A). Recordings from excitatory interneurons showed a strong bias towards outward A-type current responses (~69% for the excitatory VGLUT2+ and VGAT− neurons vs. 30% for the inhibitory VGAT+ and VGLUT2− populations, *p* < 0.001). Conversely, inhibitory interneurons were more likely to express T-type currents than excitatory populations (~38% vs. 14%, *p* < 0.001). In addition, voltage steps to −90 mV often evoked slowly activating hyperpolarization-activated cation currents in the inhibitory populations, reflecting I_h_ that was less common in excitatory populations (~78% vs. 35%, *p* < 0.001). Measurement of peak currents during steps to −40 mV (regardless of direction) and −90 mV provides an indication of the relative magnitude of these currents in each population. This comparison for the −40 mV response confirmed A-type currents dominate in excitatory populations (170 ± 203 and 169 ± 205 pA vs. −1.2 ± 96.8 and −1.7 ± 111.5 pA, for VGLUT2+ and VGAT− vs. VGAT+ and VGLUT2− cells, *p* < 0.001). This is consistent with the greater incidence of A-type potassium currents and DF AP discharge in these cells. In contrast, the lower peak current values for the inhibitory populations (VGAT+ and VGLUT2−) reflected a subset of these cells expressing pronounced inward T-type current responses (−100 to −250 pA), which is consistent with rapid burst and initial burst responses. Consistent with reports of a high incidence of TF in inhibitory populations, peak current responses during the −90 mV step (i.e., I_h_) were also larger in these cells (−26.6 ± 44.2 and −13.7 ± 25.4 pA vs. −5.8 ± 4.9 and −6.8 ± 7.3 pA, for VGAT+/VGLUT2− vs. VGLUT2+/VGAT− cells, *p* < 0.001). Thus, inhibitory dorsal horn populations tend to express voltage-gated currents that support repetitive AP discharge and TF responses dominate. In contrast, excitatory populations are more likely to express voltage-gated currents that can suppress AP discharge, with DF being more prominent in these cells.

**Figure 4:**
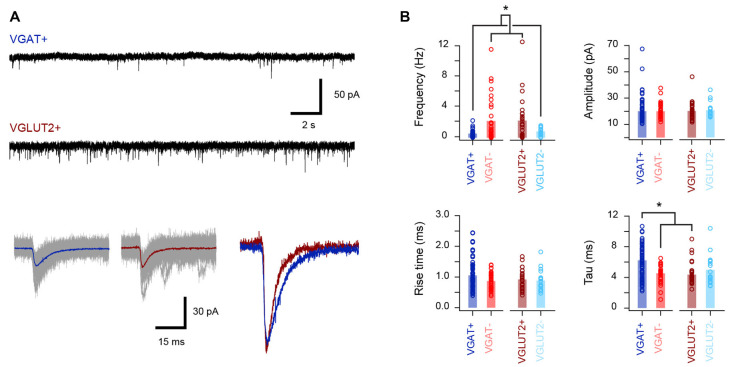
Properties of the excitatory synaptic drive to excitatory and inhibitory populations. **(A)** Upper traces show representative voltage-clamp recordings from inhibitory (vGAT+) and excitatory (vGLUT2+) neurons (holding potential −70 mV). Note the higher frequency of spontaneous excitatory postsynaptic currents (sEPSCs) in the excitatory neuron recording. Lower traces show overlaid sEPSCs captured from the above recordings with averaged currents shown as colored traces. Right traces are averages from a VGAT+ (blue) and VGLUT2+ (red) neuron scaled to the same amplitude. Note the faster sEPSCs decay kinetics in the excitatory neuron. **(B)** Group data plots comparing sEPSC properties for inhibitory and excitatory neurons. Consistent with representative traces in **(A)**, sEPSC frequency was significantly higher in excitatory neurons (VGLUT2+ and VGAT−), while sEPSC decay kinetics were slower in recordings from inhibitory cells (VGAT+ and VGLUT2−). In contrast, sEPSC amplitudes and rise times were similar across the four neuron groups. *p < 0.05.

The excitatory synaptic drive that recruits inhibitory and excitatory dorsal horn cell populations was also assessed (Figure 4, Table 4) by recording spontaneous excitatory postsynaptic currents (sEPSCs). This analysis showed that the sEPSC frequency was four times higher in the excitatory populations (VGLUT2+/VGAT− vs. VGAT+/VGLUT2− cells, *p* < 0.001), whereas the sEPSC decay time constant was slower in inhibitory interneurons (VGAT+ vs. VGLUT2+/VGAT− cells, *p* < 0.001). In contrast, sEPSC amplitude and rise time were similar across all groups (*p* = 0.99 and *p* = 0.12, respectively). Together, this is consistent with excitatory populations in LI-II receiving greater ongoing excitatory drive than inhibitory populations and, based on faster sEPSC decay times, this drive may occur at synapses located closer to their soma.

**Table 4:** Excitatory synaptic input characteristics.

Cell type	sEPSC frequency (Hz)	sEPSC peak (pA)	sEPSC rise (ms)	sEPSC Tau (ms)
VGAT+ (*targeted inhibitory*, *n* = 67)	0.41 ± 0.40* (VGAT−, VGLUT2+)	−20.33 ± 9.32	1.06 ± 0.47	6.22 ± 2.10* (VGAT−, VGLUT2+)
VGAT− (*putative excitatory*, *n* = 37)	2.06 ± 2.74* (VGAT+, VGLUT2−)	−20.09 ± 5.55	0.90 ± 0.27	4.51 ± 1.51* (VGAT+)
VGLUT2+ (*targeted excitatory*, *n* = 29)	2.11 ± 2.76* (VGAT+, VGLUT2−)	−20.44 ± 6.78	0.93 ± 0.31	4.38 ± 1.58* (VGAT+)
VGLUT2− (*putative inhibitory*, *n* = 18)	0.68 ± 0.50* (VGAT−, VGLUT2+)	−20.89 ± 5.59	0.89 ± 0.34	4.99 ± 1.95

Mean ± SD. Significant differences between groups denoted by *(bracketed below, *p* < 0.05).

### Morphological Characteristics of VGAT:Td and VGLUT2:Td Populations

**Figure 5:**
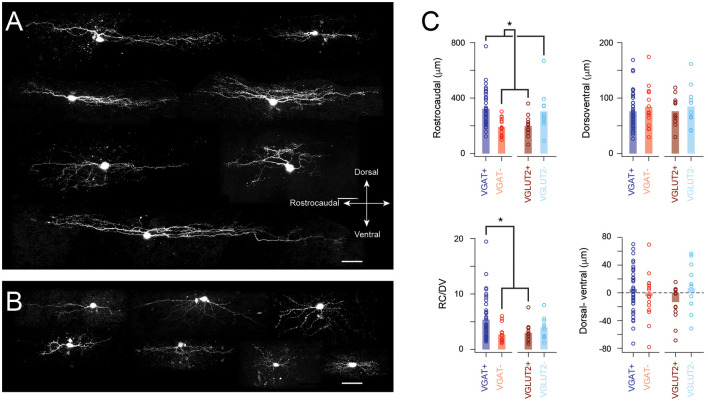
Distinct morphological features in excitatory and inhibitory populations. **(A)** Images show example morphologies from inhibitory SDH neurons. Note, these neurons exhibit extensive rostrocaudally oriented dendritic arbors. **(B)** Images show example morphologies from excitatory SDH neurons. Morphology is more diverse in this neuron population and rostrocaudal dendritic expansion is limited. **(C)** Group-data plots comparing measures of dendritic extent and orientation for inhibitory and excitatory neurons. Consistent with examples above, inhibitory neurons (VGAT+ and VGLUT2−) exhibit larger dendritic fields in the rostrocaudal plane, whereas the dorsoventral field is similar in both populations. These measurements were used to calculate a dorsoventral dendritic field ratio (RV/DV). Note, this was the largest for VGAT+ neurons compared to the excitatory population. Finally, any preference for dorsal or ventral dendritic orientation was quantified by subtracting ventral expansion from dorsal expansion. There was no difference between inhibitory and excitatory populations. Despite this, a bias in the distribution of these data was observable in the excitatory (VGLUT2+ and VGAT−) but not inhibitory (VGAT+ and VGLUT2−) datasets. This difference became significant when comparisons were limited to the VGAT+ and VGLUT2+ data (Scale bar: 50 μm). *p < 0.05.

Neurobiotin was included in recording pipettes for *post hoc* recovery and characterization of cell morphology. Interestingly, recovery was more successful in inhibitory rather than excitatory interneuron populations with yields of 55% (42/76) and 44% (11/25) for VGAT+ and VGLUT− cells, respectively (Figure 5A). In contrast, recovery yields were 35% (14/40) and 26% (15/57) for VGLUT2+ and VGAT− cells (Figure 5B). The extent of the somatodendritic tree in the rostrocaudal and dorsoventral planes was assessed for each recovered neuron (Figure 5C). Inhibitory interneurons exhibited significantly longer rostrocaudal dimensions than excitatory neurons (322 ± 124 and 304 ± 145 μm vs. 199 ± 73 and 189 ± 65 μm, VGAT+ and VGLUT2− vs. VGLUT2+ and VGAT−, *p* < 0001). In contrast, dorsoventral dimensions were similar across the four neuron types (76 ± 33 vs. 85 ± 36 vs. 76 ± 25 vs. 84 ± 38 μm, VGAT+, VGLUT2− vs. VGLUT2+, and VGAT−; *p* = 0.77).

A ratio of the rostrocaudal to dorsoventral cell dimensions (RC/DV in Figure 5C) was also calculated. This ratio was larger for the identified (VGAT+) inhibitory cells (*p* < 0.01), compared to identified and putative excitatory populations (VGLUT2+ and VGAT−). Finally, the relative bias for processes in the dorsoventral plane was assessed by calculating the difference between a cell’s ventral and dorsal extent (D-V, Figure 5C, far-right plot, both measured from the soma); a value of 0 meant cells had equivalent dorsal and ventral expansions, positive values indicated processes were more likely to extend dorsally in LI-II, whereas negative values showed processes extended ventrally (LII_i_-III). No difference was detected in dorsoventral bias across cell groups (*p* = 0.22). However, the identified excitatory interneuron group exhibited a distribution of dorsoventral measures that was heavily skewed in the ventral plane (Figure 5C, far right). Because it is likely that our putative inhibitory (VGLUT−) sample contained some excitatory interneurons, we also compared dorsoventral bias between VGAT+ and VGLUT+ cells. This showed the identified (TdTom+) excitatory population had a ventral bias compared to identified inhibitory cells (*p* = 0.04). Together, these results support the view that inhibitory dorsal horn cells exhibit extensive rostrocaudal territories, whereas excitatory interneuron processes are often biased to extend in the ventral plane.

### Heterogeneity Within VGAT:Td and VGLUT2:Td Populations

**Figure 6:**
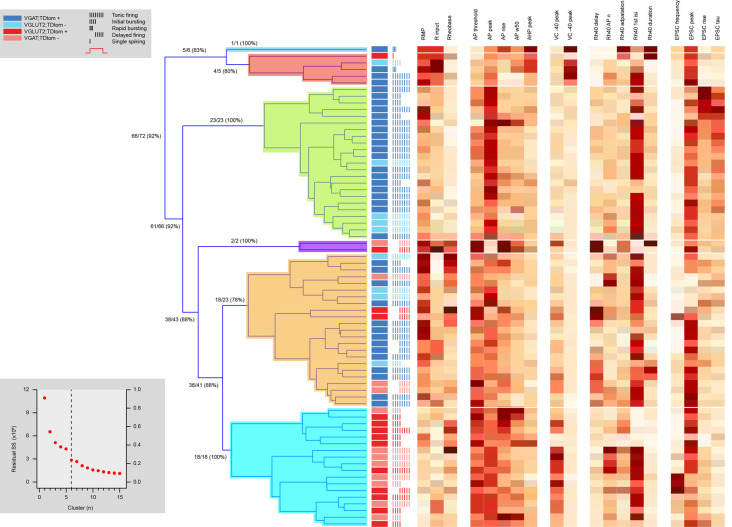
Hierarchical cluster analysis of excitatory and inhibitory dorsal horn neurons on electrophysiological attributes. Dendrogram and heatmaps plotting the assignment of dorsal horn neurons into clusters based on their electrophysiological properties. The distribution of excitatory and inhibitory neurons and their AP discharge responses are annotated using the key summarized in the upper left gray box, denoting neuron type and discharge pattern (inset). Dendrograms show the clusters (six for this analysis) determined using a K-Means elbow method, which plots the sum of squares error (SSE) for increasing cluster number to determine the point where adding another cluster does not appreciably improve the SSE (lower gray box inset). Clusters included one large inhibitory population (green cluster in dendrogram), one large excitatory population (blue), and one large mixed population of cells (orange), as well as three smaller clusters (deep blue, red, and purple). Heat maps highlight the relationship of 19 electrophysiological characteristics to each cell cluster assignment.

To further analyze cellular heterogeneity a series of hierarchical cluster analyses were performed using electrophysiological, morphological, and both sets of properties. This analysis was restricted to neurons in which we obtained both electrophysiological and morphological data (*n* = 72). Hierarchical clustering based on electrophysiological data alone yielded six distinct clusters (Figure 6). Three clusters contained many neurons (>5) whereas three were small (<5 neurons). The efficiency by which clustering segregated inhibitory and excitatory neurons was calculated by first assigning a cluster identity to the cell type that was in majority, and then determining the proportion of all neurons in this cluster that shared the same phenotype (inhibitory or excitatory). Despite some variability from cluster to cluster (78–100% cell-type purity), there was a high degree of accurate segregation, with 92% of neurons successfully clustered into their correct phenotype. Heatmaps of the properties used in this analysis showed RMP, AP threshold (AP threshold) and rise time (AP rise), voltage-activated current expression (peak at −40 peak and at −90 mV steps), and certain sEPSC properties (rise and decay time constant) were important in forming the clusters.

**Figure 7:**
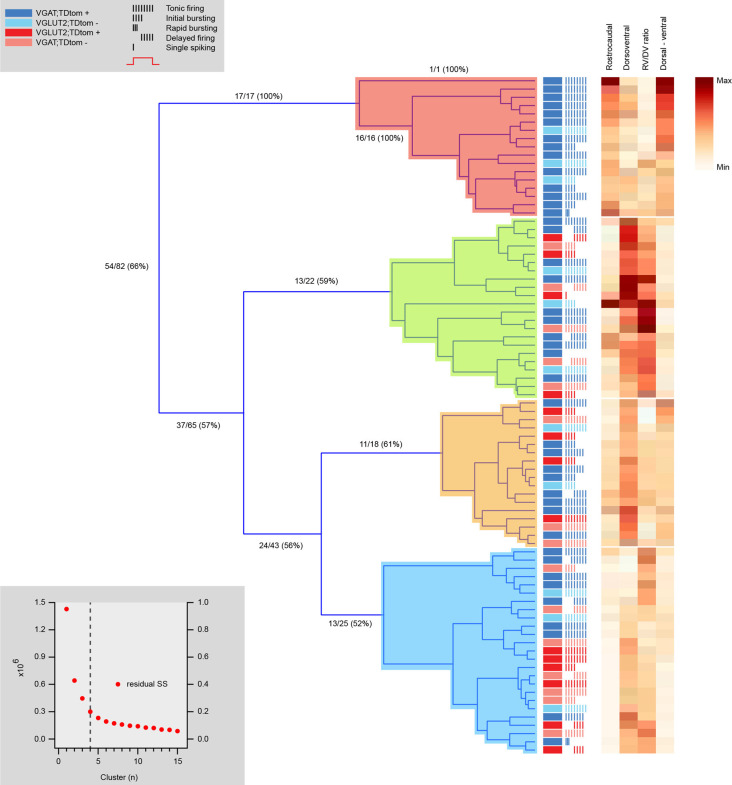
Hierarchical cluster analysis of excitatory and inhibitory dorsal horn neurons on morphological attributes. Dendrogram and heatmap showing assignment of dorsal horn neurons into clusters based on morphological properties. The distribution of excitatory and inhibitory neurons, as well as each neuron’s AP discharge response pattern, are annotated using the key summarized in the upper left gray box, denoting neuron type and discharge pattern (inset). The number of clusters (four for this analysis) was assigned using a K-Means elbow method. This method plots the sum of squares error (SSE) for increasing cluster numbers to determine when adding additional clusters does not appreciably improve the SSE (lower gray box inset). One cluster contains a population of purely inhibitory interneurons with larger rostrocaudal dendritic dimensions (red). All remaining clusters include a mix of inhibitory and excitatory neurons (green, orange, and blue). Heat maps to the right highlight the relationship of four morphological characteristics to each cell cluster assignment.

Hierarchical clustering of the same 72 neurons according to morphological features segregated four distinct clusters with similar proportions of cells (Figure 7). One cluster contained purely inhibitory neurons, while the remaining three clusters contained a mix of inhibitory and excitatory neurons. Thus, morphology-based clustering did not segregate neurons as well as our electrophysiological analysis (Figure 6), with only 66% of neurons segregating with the same phenotype. This suggests electrophysiological properties better discriminate between the excitatory and inhibitory cell phenotypes, however, fewer properties were used in the morphology vs. electrophysiology analysis (4 vs. 19). Regardless, the heatmaps of the four dendritic dimension measurements (rostrocaudal length, dorsoventral length, rostrocaudal/dorsoventral ratio, and dorsoventral bias) suggest each parameter contributed to segregation.

**Figure 8:**
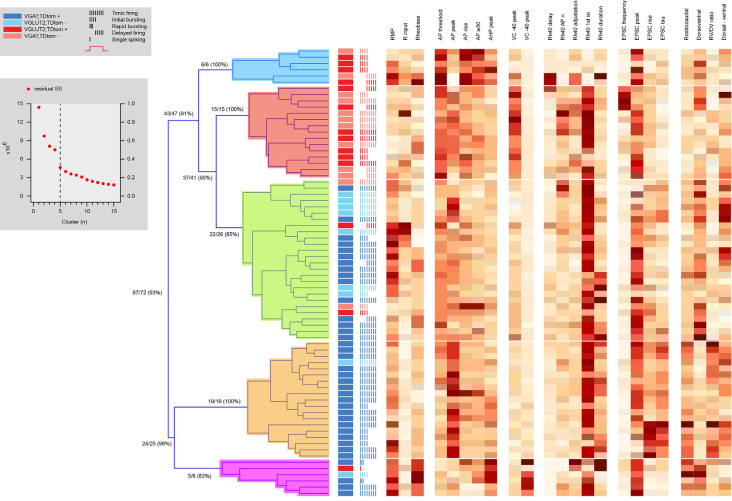
Hierarchical cluster analysis of excitatory and inhibitory dorsal horn neurons on combined electrophysiological and morphological attributes. Dendrogram and heatmap plotting the assignment of dorsal horn neurons into clusters based on their electrophysiological and morphological properties. The distribution of excitatory and inhibitory neurons, as well as the AP discharge response pattern for each neuron, are annotated using the key summarized in the lower-left gray box, denoting neuron type and discharge pattern (inset). The number of clusters (four for this analysis) was assigned using a K-Means elbow method that plots the sum of squares error (SSE) for increasing cluster number to determine the point where additional clusters do not appreciably improve the SSE (upper gray box inset). Clustering using both electrophysiological and morphological attributes differentiates two purely excitatory clusters (blue and red), one purely inhibitory cluster (orange), and two clusters also dominated by inhibitory cells (green and pink). Heat maps highlight the relationship of 19 electrophysiological, and four morphological characteristics to each cell cluster assignment.

Hierarchical clustering combining electrophysiological and morphological features yielded five distinct clusters (Figure 8, Table 5), two excitatory groups (Ex1 and Ex2) and three inhibitory groups (In1, In2, and In3). Only two of the inhibitory clusters contained any excitatory cells. The addition of morphological features did not appreciably improve clustering accuracy (93% of neurons segregating with the same phenotype, vs. 92% in electrophysiology only clustering). Heatmaps also showed the same properties that influenced independent electrophysiological or morphological clustering also did so in the combined clustering analysis. Table 5 highlights a range of properties that differed across clusters, not surprising given the nature of the hierarchical clustering approach. Also predictable, given the differences between excitatory and inhibitory interneurons (Tables s 1, 2, 4; and Figures 2–5), many properties differed between excitatory vs. inhibitory clusters. Most interesting, however, were properties that distinguished within the excitatory or inhibitory clusters. AP properties including peak amplitude, rise and half-width, as well as the duration of spiking and number of spikes discharged all differed between excitatory clusters (Ex1 vs. Ex2). In addition, peak A-current (VC −40 mV peak) and sEPSC frequency also differed between the excitatory clusters. For inhibitory clusters, Rheobase current, AP and AHP peak, Ih current peak (VC -90 mV peak), the first AP interspike interval (1st AP ISI), AP adaptation, sEPSC time course (rise time and Tau), and morphological features (rostrocaudal extent, dorsoventral extent, and the RC:DV ratio) distinguished clusters (In1 vs. In2 vs. In3).

**Table 5:** Cluster specific properties.

	Cluster 1 (Ex1)	Cluster 2 (Ex2)	Cluster 3 (In1)	Cluster 4 (In2)	Cluster 5 (In3)
RMP (mV)	−60.22 ± 11.58	−71.28 ± 5.20 *In1, In2, In3	−57.64 ± −10.20 *Ex2	−61.60 ± 8.55 *Ex2	−50.69 ± 3.950 *Ex2
Rinput (MΩ)	268.04 ± 94.98	257.49 ± 136.12 *In2	243.68 ± 159.52 *In2	116.83 ± 28.80 *Ex2, In1	144.86 ± 99.012
Rheobase (pA)	60.00 ± 35.78 *In3	45.33 ± 20.66 *In3	45.38 ± 19.23 *In3	46.32 ± 13.42 *In3	100.00 ± 17.89 *Ex1, Ex2, In1, In2
AP threshold (mV)	−21.26 ± 5.75 *In1, In2, In3	−26.43 ± 3.25 *In1, In2, In3	−30.77 ± 3.23 *Ex1, Ex2, In3	−33.58 ± 4.15 *Ex1, Ex2	−37.74 ± 3.18 *Ex1, Ex2, In1
AP peak (mV)	42.30 ± 18.05 *Ex2, In1, In2	58.78 ± 9.68 *Ex1, In2	57.30 ± 9.54 *Ex1, In2	72.00 ± 7.00 *Ex1, Ex2, In1, In3	47.94 ± 6.83 *In2
AP rise (ms)	1.40 ± 0.29 *Ex2, In1, In2, In3	0.99 ± 0.145 *Ex1	0.87 ± 0.19 *Ex1	0.87 ± 0.18 *Ex1	0.77 ± 0.27 *Ex1
AP w50 (ms)	5.17 ± 1.17 *Ex2, In1, In2, In3	3.61 ± 0.43 *Ex1	3.10 ± 0.73 *Ex1	3.41 ± 0.53 *Ex1	3.33 ± 1.01 *Ex1
AHP peak (mV)	−15.34 ± 6.85 *In1	−23.42 ± 4.51 *In3	−23.96 ± 3.39 *Ex1, In3	−20.76 ± 5.19 *In3	−6.11 ± 11.13 *Ex2, In1, In2
VC −40 mV peak (mV)	50.03 ± 184.73 *Ex2	356.82 ± 162.81 *Ex1, In1, In2, In3	34.99 ± 110.61 *Ex2	−26.61 ± 61.69 *Ex2	9.99 ± 44.36 *Ex2
VC −90 mV peak (mV)	−3.43 ± 1.47 *In3	−6.15 ± 3.63 *In3	−6.72 ± 5.50 *In3	−16.15 ± 13.79 *In3	−119.92 ± 67.64 *Ex1, Ex2, In1, In2
AP delay (ms)	106.30 ± 114.65 *In3	53.09 ± 49.44	52.10 ± 34.06	43.51 ± 11.77	13.37 ± 3.88 *Ex1
1st AP ISI (ms)	148.87 ± 287.51	40.57 ± 9.87 *In3	60.29 ± 37.33 *In3	63.01 ± 16.70 *In3	279.93 ± 407.22 *Ex2, In1, In2
AP duration (ms)	132.38 ± 116.80 *Ex2, In1, In2, In3	786.07 ± 233.29 *Ex1	816.68 ± 157.41 *Ex1	784.15 ± 208.03 *Ex1	633.77 ± 349.83 *Ex1
APs (*n*)	3.17 ± 1.33 *Ex2	15.33 ± 7.86 *Ex1, In2	11.15 ± 6.04	8.95 ± 3.24 *Ex2	8.00 ± 6.20
AP spiking adaptation	0.60 ± 0.38	0.63 ± 0.34	0.45 ± 0.18 *In3	0.45 ± 0.12 *In3	3.02 ± 6.14 *In1, In2
sEPSC frequency (Hz)	0.51 ± 0.41 *Ex2	2.65 ± 2.59 *Ex1, In1, In2	0.585 ± 0.44 *Ex2	0.43 ± 0.45 *Ex2	1.01 ± 0.57
sEPSC peak (pA)	−17.53 ± 6.10	−18.52 ± 4.21	−19.32 ± 6.56	−19.61 ± 5.32	−15.31 ± 2.64
sEPSC rise (ms)	0.77 ± 0.27 *In2	0.91 ± 0.19 *In2	1.00 ± 0.29 *In2	1.43 ± 0.54 *Ex1, Ex2, In1, In3	0.61 ± 0.30 *In2
sEPSC tau (ms)	4.85 ± 1.01 *In2	3.71 ± 0.73 *In1, In2	5.27 ± 1.55 *Ex2, In2	7.97 ± 1.86 *Ex1, Ex2, In1, In3	3.97 ± 0.65 *In2
Rostrocaudal (μm)	217.71 ± 68.86 *In2, In3	169.30 ± 55.87 *In2, In3	245.76 ± 75.50 *In2, In3	381.71 ± 130.52 *Ex1, Ex2, In1	413.18 ± 172.95 *Ex1, Ex2, In1
Dorsoventral (μm)	79.50 ± 23.97	64.58 ± 23.97 *In1, In3	90.72 ± 26.81 *Ex2, In2	48.58 ± 11.67 *In1, In3	101.62 ± 33.07 *Ex2, In2
RC:DV	2.87 ± 1.02 *In2	3.19 ± 2.02 *In2	2.92 ± 1.19 *In2	8.36 ± 3.76 *Ex1, Ex2, In1, In3	4.167 ± 1.65 *In2
Dorsal—ventral (μm)	−11.76 ± 34.76	−1.52 ± 28.38	3.73 ± 37.21	10.99 ± 21.17	−13.22 ± 37.98

*p < 0.05.

**Figure 9:**
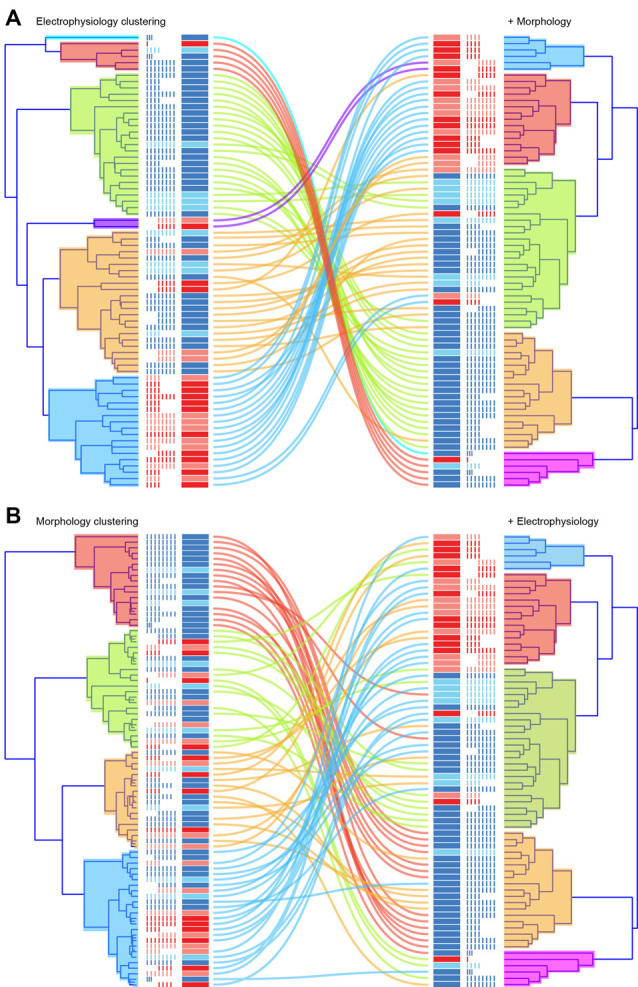
Correlation of cluster membership across clustering approaches. The figure compares individual cell cluster membership when clustering is based on electrophysiological (**A**, upper left) or morphological data (**B**, lower left), and when both datasets are combined (right for both **A,B**). Corresponding dendrograms are presented side-by-side, with lines denoting cell location in clustering (left vs. right). When the path of a colored line remains close to others from its original cluster the clusters are shared: electrophysiological vs. electrophysiological plus morphological clustering **(A)**; or morphological vs. morphological plus electrophysiological clustering **(B)**. Note cluster membership remains similar in **(A)** but diversifies more dramatically in (**B**; i.e., there is more mixing of the colored lines).

Finally, we compared how cluster membership for individual neurons changed between analyses based on electrophysiological, morphological or combined morphological and electrophysiological datasets (Figure 9). For the electrophysiological approach (Figure 9A), most cells from each of the original six clusters remained grouped together when morphology data were added (100%, 100%, 78%, 100%, 83%, 67%). For excitatory neurons, one large cluster split into two distinct clusters and a smaller population merged with one of these groups. Inhibitory neurons, which originally segregated into two large and two small clusters, remained grouped together with some cells switching or merging to form three distinct clusters. For the morphological approach, inhibitory neurons from one original cluster stayed tightly grouped (81%), while two other mixed cell clusters (excitatory and inhibitory neuron membership) split, contributing to each of the five clusters that were resolved when electrophysiological data was added. Not surprisingly, given the initial cluster analysis results, the variety of electrophysiological attributes resolved relatively pure clusters in terms of correct assignment of excitatory and inhibitory phenotypes. While the addition of morphological features did not change cluster purity, it did further distinguish these groups providing an enhanced view of this heterogeneous population.

## Discussion

Resolving functionally discrete populations of neurons that serve specific roles in neuronal circuits remains a major challenge in neuroscience (Zeng and Sanes, 2017). This is certainly true of the dorsal horn where neuronal heterogeneity is substantial, and knowledge of how cell populations are assembled into circuits is essential to understand the normal sensory experience. Historically, a variety of approaches have been used to dissect these heterogeneous populations including electrophysiological properties, morphology, and neurochemical content (Grudt and Perl, 2002; Graham et al., 2004, 2008; Yasaka et al., 2010; Gutierrez-Mecinas et al., 2016; Boyle et al., 2017). Recent single-cell molecular techniques, however, have provided an alternative approach to neuron classification and, in the dorsal horn, suggested many more discrete subpopulations exist than predicted by the historical literature. This prompted the current study.

Our results highlight a number of electrophysiological properties that distinguish excitatory or inhibitory dorsal horn neurons, though these properties do not segregate entirely to either population. When a suite of electrophysiological parameters are used to perform a hierarchical cluster analysis, excitatory and inhibitory interneurons reliably segregate into relatively pure cluster groups (with 92% purity, Figure 5). In contrast, the same analysis on morphological features yielded poorer clustering (66% purity, Figure 6), though one cluster did contain only inhibitory cells and most likely represents the well-established islet-inhibitory phenotype. When electrophysiological and morphological features were combined, clustering was similar to using electrophysiological properties alone, discriminating five distinct groups; two excitatory and three inhibitory. Our findings have a number of implications for our views of dorsal horn neuron heterogeneity and they also raise a number of questions and caveats for future studies.

## Limitations

Although the use of transgenic animals to prospectively identify excitatory and inhibitory populations has obvious advantages, it also has limitations. First, our immunolabeling indicated that transgenically labeled populations (*via* Tdtomato expression) reliably marked excitatory and inhibitory cells (>90% of excitatory and inhibitory cells correctly labeled). Thus, we can be confident that most targeted recordings and subsequent morphological analysis assessed the intended excitatory or inhibitory cell types. We also collect data from unlabeled cells in each transgenic line under the premise that these cells should represent the opposing population (e.g., presumptive inhibitory cells in the VGLUT2:Td mouse). This assumption appears to largely hold with the VGAT:Td line as TdTom labeled 87% of inhibitory cells (assessed by co-labeling for Pax2) leaving a fraction of the VGAT−Td negative cells (~13%) were also inhibitory. Thus, recordings from VGAT− cells, assumed to be excitatory neurons, may include some inhibitory cells.

Contrasting the relatively high capture of inhibitory interneurons in VGAT:Td mice, we estimate that the TdTom labeled approximately a third of the excitatory interneurons in VGLUT2:Td mice. This data did not allow us to determine if the TdTom−labeled population represented a cross-section of excitatory dorsal horn neurons or a discrete subset. The similarity in the values and distribution of electrophysiological and morphological data between the VGLUT+ and VGAT− samples favors the likelihood that the VGLUT2+ sample captures a cross-section of these cells. Future work screening a panel of neurochemical (Gutierrez-Mecinas et al., 2016) or molecular (Haring et al., 2018) markers known to be restricted to excitatory populations will be necessary to further clarify this point.

Another implication of the lower labeling yield in VGLUT2:Td mice is the potential TdTom−negative recordings included both inhibitory and excitatory interneurons. Despite this caveat, many of the comparisons between VGAT+ and VGLUT2− cells are strikingly similar. For example, the similar distributions of AP discharge patterns and spontaneous EPSC frequency in VGAT+/VGLUT2− compared to the VGLUT2+/VGAT− data (Figures 2, 4). A number of factors may have contributed to this result, including a difference in identification and sampling of TdTom−labeling in fixed tissue vs. endogenous TdTom−expression in fresh spinal slices. Specifically, immunolabeling enhanced the fluorescence signal in fine dendrites and axons in fixed tissue. This effectively increased background labeling in the dense VGLUT2+ plexus of the dorsal horn, making it challenging to resolve weakly labeled somatic profiles. Alternatively, fluorescent TdTom−signals in fresh tissue could only be resolved in cell somas, and exposure was varied to verify the presence or absence of even weak signals. Thus, the populations described in fixed tissue samples may not be identical to those recorded in fresh slices. In addition, an inherent unintended bias when selecting cells to record in slice (always present in patch-clamp recordings) may have influenced our sampling across mouse lines (Smith et al., 2015). Regardless, some data still points to contamination of the VGLUT2-negative sample by excitatory cells, such as an elevated incidence of A-type potassium currents (~45%), which is widely regarded as a property restricted to excitatory populations. Altogether, these caveats informed our use of the terms “identified” excitatory/inhibitory neurons for VGAT+/VGLUT2-samples and “putative” excitatory/inhibitory neurons for VGAT−/VGLUT2− data. These considerations will be important for future studies employing the VGAT-cre and VGLUT2-cre animals to study populations in the dorsal horn.

Our targeted recording approach allowed us to obtain similar sample sizes for excitatory and inhibitory neurons. While this was a statistically appropriate approach, it is at odds with the established ratio of approximately 3:1 for excitatory and inhibitory populations (Harris et al., 2018; Sathyamurthy et al., 2018), and thus introduces a sampling bias that may have influenced our clustering results. Similarly, our cluster analysis was restricted to cells that yielded both electrophysiological and morphology data. This limited our overall sample size for cluster analyses. These factors likely affected our capacity to confidently resolve smaller subpopulations of excitatory and inhibitory neurons. Future work that expands these samples may resolve a greater degree of functional and morphological heterogeneity. Also regarding clustering, we used the K-means elbow method to determine the number of clusters within our data, which is a conservative method. Thus, our data may contain additional electrophysiologically and morphologically distinct populations obscured by this approach. This is supported by the heatmap data for the hierarchical clustering (Figures 6–8), which contains a number of AP, sEPSC, and morphological parameters that are distinctly distributed with clear boundaries, even within clusters. Larger sample sizes could address this issue, as well as the inclusion of comparable data from other genetically identified populations. For example, incorporating electrophysiological and morphological data from somatostatin, dynorphin, PV, transient VGLUT3, and calretinin expressing populations would test whether these well-characterized populations cluster discretely, or if they overlap with the identified excitatory and inhibitory populations in the current study.

### Distinguishing Excitatory and Inhibitory Interneuron Characteristics

A recent study relates closely to the excitatory vs. inhibitory dataset in our experiments (Punnakkal et al., 2014). Using a Bac-transgenic mouse, which labeled VGLUT2-positive neurons with a green fluorescent protein (VGLUT2:GFP), a variety of electrophysiological and morphological properties were compared with two other commonly used transgenic lines that label GABAergic (GAD67:GFP) and glycinergic (GlyT2:GFP) interneurons, respectively. A number of electrophysiological properties were identified that differentiated the VGLUT2:GFP population from GAD67:GFP and GlyT2:GFP recordings. For example, DF AP discharge was more prevalent in VGLUT2:GFP recordings, whereas TF dominated in the GAD67:GFP and GlyT2:GFP samples. This work also highlighted a larger mean rheobase current, a more depolarized AP threshold, and broader APs for the VGLUT2:GFP neurons. The anatomy of VGLUT2:GFP neurons showed that these cells had central, radial and vertical, but never islet cell morphologies. Our data broadly agree with this study, though we did identify differences in properties such as input resistance, RMP, AP peak, and AP rise time not reported in the previous study. Likewise, we did not detect differences in rheobase current. While some of these disparities may be due to technical differences, both studies highlight that each transgenic line did not identify all VGLUT2+ neurons with TdTomato (~30%) or GFP (20–40%), respectively. Thus, datasets may overlap, without necessarily sampling the same population. Regardless, the similarity of our results and this work provide confidence that the VGLUT2:Td mouse line provides an opportunity to better understand heterogeneity within dorsal horn populations.

Another study has sought to clarify the inhibitory nature of labeled VGAT-positive cells, before going on to manipulate these cells and monitor behavioral outcomes (Koga et al., 2017). This work compared VGAT-RFP expression with total Pax2 and showed that the labeled vGAT+ cells were almost all inhibitory (~98% of vGAT+ were Pax2+). The proportion of all inhibitory cells captured was, however, much smaller (~25% Pax2 overlapped with the vGAT+) and most likely reflects sparse viral labeling of the VGAT-Cre population in contrast to our transgenic breeding approach. Some targeted recordings (*n* = 5) were made in this study and showed that all recorded cells exhibited the TF discharge pattern that is typical of inhibitory interneurons. While the majority of vGAT+ cells (~75%) in our study exhibited TF, there was more diversity including; initial/rapid bursting (~18%), delayed (5%) and single spiking (1%) patterns. This suggests we captured a more heterogeneous population, which can only be uncovered in a larger sample size (*n* = 5 vs. *n* = 74, respectively). Nevertheless, these data show a VGAT−Tdtom mouse is a useful tool for teasing apart, from and inhibitory standpoint, the complex circuitry of the dorsal horn.

Other studies have also undertaken electrophysiological and morphological characterizations of identified dorsal horn neurons (Hantman et al., 2004; Heinke et al., 2004; Zeilhofer et al., 2005; Hughes et al., 2012; Duan et al., 2014; Punnakkal et al., 2014; Smith et al., 2015; Dickie et al., 2019). Together with the above work, they provide a number of insights into the features that distinguish different subpopulations. For example, TF AP discharge is associated with inhibitory populations (Hantman et al., 2004; Heinke et al., 2004; Zeilhofer et al., 2005; Smith et al., 2015), whereas DF is usually associated with excitatory cell types (Punnakkal et al., 2014; Smith et al., 2015; Dickie et al., 2019). Consistent with these observations, the hyperpolarization-activated cation current (Ih), which supports repetitive AP discharge, is common among inhibitory populations. The A-type potassium current, on the other hand, delays AP discharge and is typically expressed in excitatory dorsal horn neurons (Punnakkal et al., 2014; Smith et al., 2015; Dickie et al., 2019). We, and others, have also noted that inhibitory dorsal horn neurons receive low frequency spontaneous excitatory input, whereas excitatory interneurons often receive much higher frequency input. The data presented here agree with these observations but also cautions against using these properties to distinguish inhibitory and excitatory populations. For example, the TF was also observed among subsets of excitatory neurons, A-type currents were observed in some inhibitory interneurons, and not all excitatory cells received high frequency spontaneous excitatory input.

In addition to the above differences, a number of other properties differed in recordings from inhibitory and excitatory dorsal horn neurons. Specifically, inhibitory neurons had lower input resistances, more depolarized membrane potentials, hyperpolarized AP threshold, larger AP peak amplitude, faster AP kinetics (rise and half-width), and slower sEPSC decay kinetics than the excitatory population. Many of these properties are consistent with the more excitable electrophysiological phenotype of TF inhibitory interneurons. In addition, some of these differences likely relate to the distinct morphological features of inhibitory and excitatory neurons in the dorsal horn. Specifically, our inhibitory sample showed much longer rostrocaudal dendritic profiles, whereas dorsoventral arborizations were similar to the excitatory sample. The larger dendritic trees are consistent with the lower input resistance of our inhibitory neurons (Barrett and Crill, 1974) and that distal excitatory synaptic inputs in this population have slower decay time courses (Rall et al., 1967).

Our morphological observations are also consistent with the well-established morphological categories for classifying dorsal horn neurons in lamina II (Grudt and Perl, 2002; Maxwell et al., 2007; Yasaka et al., 2010), where many of our recordings were made. Lamina II cells that exhibit expansive dendritic arbors in the rostrocaudal plane are classified as having islet morphology, and this appearance is reliably associated with inhibitory cell phenotypes. By contrast, cells with compact dendritic fields extending in all planes (i.e., radial cell morphology) are associated with an excitatory phenotype. Likewise, cells with a distinct bias towards ventrally projecting dendrites, often emanating from a common dendrite, are termed vertical cells and are typically excitatory, though these characteristics have also been described in some inhibitory cells. Finally, dorsal horn neurons with rostrocaudally oriented dendritic arbors like the islet category, but smaller in overall size, are classified as central cells and are observed in both excitatory and inhibitory populations. Our results on morphology, with longer rostrocaudal spread and larger rostrocaudal to dorsoventral ratios for dendrites in inhibitory interneurons, as well as the ventral bias of dendrites in excitatory interneurons, agree with this literature.

Despite the above associations, we did not classify the morphology of recorded cells in this study for two reasons. First, the morphological classification described above was developed specifically for neurons in lamina II, however, our recordings sampled a region that would include lamina I cells and potentially some in lamina III. Precisely locating cells to laminae require additional immunolabeling to confidently identify laminae borders. The second reason relates to cells that do not fit the criteria for this classification. In most studies that classify dorsal horn neurons in this way, an additional group of unclassified neurons is typically reported (Grudt and Perl, 2002; Heinke et al., 2004; Maxwell et al., 2007; Yasaka et al., 2010). Hence, to avoid omission of cells we simply reported and compared pertinent morphological characteristics and tested the ability of these properties to provide insight on neuronal heterogeneity. Interestingly, although differences were detected between the two populations (excitatory and inhibitory) in group comparisons, the hierarchical cluster analysis showed that morphological parameters did not efficiently separate excitatory and inhibitory populations into discrete clusters (Figure 7, 66% cluster purity). This is not surprising, as widely-accepted anatomical schema correlating morphologies (other than islet) to an excitatory or inhibitory phenotype is still elusive (Grudt and Perl, 2002; Heinke et al., 2004). This lack of cluster discrimination, however, may also reflect the limited dataset used for this analysis, which only used four morphological properties. This contrasts with clustering on the basis of electrophysiological properties (Figure 6), where 19 properties were used and yielded a cluster purity of 92%. Finally, as noted above a relatively small sample size will have constrained the capacity of this approach to distinguish clusters. This was influenced by the difficulty in obtaining sufficient morphological recoveries from small interneuron populations in the dorsal horn, reducing the data included for cluster analysis. Morphological recovery data also reinforced a developing view that excitatory dorsal horn neurons are more difficult to fill and recover during patch-clamp experiments than inhibitory cell types. The current dataset, which recorded (and attempted to recover morphology) from both excitatory and inhibitory populations using identical conditions provides quantitative data to support this view, with a recovery rate of 53% for inhibitory cells but only 30% for excitatory cells. This identifies a technical bias that potentially influences sampling in previous studies that rely on neurobiotin recovery to characterize dorsal horn populations. Furthermore, it suggests that transgenic sparse labeling approaches (Jefferis and Livet, 2012), or the use of rainbow labeling techniques (Cai et al., 2013) will be required to properly survey the morphological properties of these populations.

## Conclusions

The two transgenic lines utilized throughout this study, vGAT:Td and vGLUT2:TdTom, positively identify inhibitory and excitatory cells and are thus useful for studying these broad cell populations. Furthermore, in cross-referencing the electrophysiological and morphological properties of these excitatory and inhibitory neurons in the SDH, our experiments support the conclusions of several previous studies characterizing various dorsal horn subpopulations. Inhibitory interneurons in this region are generally more excitable and easily recruited with TF discharge responses dominating, while the ongoing excitatory synaptic drive to this population is weak. This population also exhibits extensive rostrocaudal dendritic arborizations and features synaptic inputs on their distal dendrites that undergo substantial filtering. In contrast, excitatory interneurons are generally less excitable with a greater degree of DF and IB discharge profiles, and excitatory synaptic drive to this population is strong. This population exhibits a general bias in their dendritic arbors in the ventral plane, consistent with dorsal horn models that assign excitatory interneurons a role in transmitting signals from ventral to dorsal aspects of this region (Torsney and MacDermott, 2006). An important caveat to these findings is that although the above overall associations were clear, most of the properties highlighted also occurred (on occasion) in the opposing population. This cautions against using electrophysiological characteristics or morphology to differentiate excitatory and inhibitory cells and suggests the collection of multiple electrophysiological characteristics is a more accurate approach. The other key finding from these comparisons is that while as many as 30 different molecular classes of dorsal horn neurons have been differentiated (Haring et al., 2018), only five relatively distinct groups can be differentiated on electrophysiological and morphological grounds. A number of considerations may help to reconcile these differences. First, there may be some level of phenotypic convergence in the properties we assessed among the molecular classes (Konstantinides et al., 2018). Assessing additional properties such as inhibitory input (Smith et al., 2016) and connectivity (Kosugi et al., 2013; Hachisuka et al., 2018) may help to further differentiate the functional classes we identified. Further, increasing the sample size for our data, incorporating recordings from other transgenic mouse lines to identify additional distinct subpopulations (Hantman et al., 2004; Heinke et al., 2004; Zeilhofer et al., 2005; Hughes et al., 2012; Duan et al., 2014; Peirs et al., 2015; Smith et al., 2015; Hachisuka et al., 2018; Dickie et al., 2019), and balancing the relative ratio of excitatory to inhibitory recordings may help clustering methods to differentiate functionally distinct dorsal horn neurons classes. Ultimately, however, a unifying view of the subpopulations of dorsal horn neurons that play unique roles in function is likely to require integration of molecular, functional and connectomic approaches to heterogeneity. The data presented here represents one step towards this process.

## Data Availability Statement

All datasets generated for this study are included in the article.

## Ethics Statement

The animal study was reviewed and approved by The University of Newcastle Animal Care and Ethics Committee under Section 25 of the NSW Animal Research Act, 1985.

## Author Contributions

TB, CD, RC, DH, and BG conceived and designed the research study. TB, MG, and JI conducted experiments and acquired data. TB, MG, JI, JM, DH, and BG analyzed data. BG, TB, and RC wrote the manuscript. All authors edited the final version of the manuscript.

## Conflict of Interest

The authors declare that the research was conducted in the absence of any commercial or financial relationships that could be construed as a potential conflict of interest.

## References

[B1] Alba-Delgado C. El Khoueiry C. Peirs C. Dallel R. Artola A. Antri M. (2015). Subpopulations of PKCγ interneurons within the medullary dorsal horn revealed by electrophysiologic and morphologic approach. Pain 156, 1714–1728. 10.1097/j.pain.0000000000000221 25961142

[B2] Barrett J. N. Crill W. E. (1974). Specific membrane properties of cat motoneurones. J. Physiol. 239, 301–324. 10.1113/jphysiol.1974.sp010570 4137933 PMC1330925

[B3] Bourane S. Duan B. Koch S. C. Dalet A. Britz O. Garcia-Campmany L.. (2015). Gate control of mechanical itch by a subpopulation of spinal cord interneurons. Science 350, 550–554. 10.1126/science.aac8653 26516282 PMC4700934

[B4] Boyle K. A. Gradwell M. A. Yasaka T. Dickie A. C. Polgar E. Ganley R. P.. (2019). Defining a spinal microcircuit that gates myelinated afferent input: implications for tactile allodynia. Cell Rep. 28, 526.e6–540.e6. 10.1016/j.celrep.2019.06.040 31291586 PMC6635381

[B5] Boyle K. A. Gutierrez-Mecinas M. Polgár E. Mooney N. O’Connor E. Furuta T.. (2017). A quantitative study of neurochemically defined populations of inhibitory interneurons in the superficial dorsal horn of the mouse spinal cord. Neuroscience 363, 120–133. 10.1016/j.neuroscience.2017.08.044 28860091 PMC5648048

[B6] Braz J. Solorzano C. Wang X. Basbaum A. I. (2014). Transmitting pain and itch messages: a contemporary view of the spinal cord circuits that generate gate control. Neuron 82, 522–536. 10.1016/j.neuron.2014.01.018 24811377 PMC4492533

[B7] Cai D. Cohen K. B. Luo T. Lichtman J. W. Sanes J. R. (2013). Improved tools for the Brainbow toolbox. Nat. Methods 10, 540–547. 10.1038/nmeth.2450 23817127 PMC3713494

[B8] Cameron D. Polgár E. Gutierrez-Mecinas M. Gomez-Lima M. Watanabe M. Todd A. J. (2015). The organisation of spinoparabrachial neurons in the mouse. Pain 156, 2061–2071. 10.1097/j.pain.0000000000000270 26101837 PMC4770364

[B9] Cauli B. Porter J. T. Tsuzuki K. Lambolez B. Rossier J. Quenet B.. (2000). Classification of fusiform neocortical interneurons based on unsupervised clustering. Proc. Natl. Acad. Sci. U S A 97, 6144–6149. 10.1073/pnas.97.11.6144 10823957 PMC18572

[B560] Demšar J. Curk T. Erjavec A. Gorup C. Hocevar T. Milutinovic M.. (2013). Orange: data mining toolbox in python. J. Machine Learn. Res. 14, 2349–2353. 15611994

[B10] Dickie A. C. Bell A. M. Iwagaki N. Polgár E. Gutierrez-Mecinas M. Kelly R.. (2019). Morphological and functional properties distinguish the substance P and gastrin-releasing peptide subsets of excitatory interneuron in the spinal cord dorsal horn. Pain 160, 442–462. 10.1097/j.pain.0000000000001406 30247267 PMC6330098

[B11] Duan B. Cheng L. Bourane S. Britz O. Padilla C. Garcia-Campmany L.. (2014). Identification of spinal circuits transmitting and gating mechanical pain. Cell 159, 1417–1432. 10.1016/j.cell.2014.11.003 25467445 PMC4258511

[B12] Foster E. Wildner H. Tudeau L. Haueter S. Ralvenius W. T. Jegen M.. (2015). Targeted ablation, silencing, and activation establish glycinergic dorsal horn neurons as key components of a spinal gate for pain and itch. Neuron 85, 1289–1304. 10.1016/j.neuron.2015.02.028 25789756 PMC4372258

[B13] François A. Low S. A. Sypek E. I. Christensen A. J. Sotoudeh C. Beier K. T.. (2017). A brainstem-spinal cord inhibitory circuit for mechanical pain modulation by GABA and enkephalins. Neuron 93, 822.e6–839.e6. 10.1016/j.neuron.2017.01.008 28162807 PMC7354674

[B14] Gouwens N. W. Sorensen S. A. Berg J. Lee C. Jarsky T. Ting J.. (2019). Classification of electrophysiological and morphological neuron types in the mouse visual cortex. Nat. Neurosci. 22, 1182–1195. 10.1038/s41593-019-0417-0 31209381 PMC8078853

[B15] Graham B. A. Brichta A. M. Callister R. J. (2004). *In vivo* responses of mouse superficial dorsal horn neurones to both current injection and peripheral cutaneous stimulation. J. Physiol. 561, 749–763. 10.1113/jphysiol.2004.072645 15604230 PMC1665382

[B16] Graham B. A. Brichta A. M. Callister R. J. (2008). Recording temperature affects the excitability of mouse superficial dorsal horn neurons, *in vitro*. J. Neurophysiol. 99, 2048–2059. 10.1152/jn.01176.2007 18287548

[B17] Graham B. A. Brichta A. M. Schofield P. R. Callister R. J. (2007). Altered potassium channel function in the superficial dorsal horn of the spastic mouse. J. Physiol. 584, 121–136. 10.1113/jphysiol.2007.138198 17690143 PMC2277054

[B18] Graves A. R. Moore S. J. Bloss E. B. Mensh B. D. Kath W. L. Spruston N. (2012). Hippocampal pyramidal neurons comprise two distinct cell types that are countermodulated by metabotropic receptors. Neuron 76, 776–789. 10.1016/j.neuron.2012.09.036 23177962 PMC3509417

[B19] Grudt T. J. Perl E. R. (2002). Correlations between neuronal morphology and electrophysiological features in the rodent superficial dorsal horn. J. Physiol. 540, 189–207. 10.1113/jphysiol.2001.012890 11927679 PMC2290200

[B20] Gutierrez-Mecinas M. Davis O. Polgár E. Shahzad M. Navarro-Batista K. Furuta T.. (2019). Expression of calretinin among different neurochemical classes of interneuron in the superficial dorsal horn of the mouse spinal cord. Neuroscience 398, 171–181. 10.1016/j.neuroscience.2018.12.009 30553791 PMC6347472

[B21] Gutierrez-Mecinas M. Furuta T. Watanabe M. Todd A. J. (2016). A quantitative study of neurochemically defined excitatory interneuron populations in laminae I-III of the mouse spinal cord. Mol. Pain 12:1744806916629065. 10.1177/1744806916629065 27030714 PMC4946630

[B22] Hachisuka J. Omori Y. Chiang M. C. Gold M. S. Koerber H. R. Ross S. E. (2018). Wind-up in lamina I spinoparabrachial neurons: a role for reverberatory circuits. Pain 159, 1484–1493. 10.1097/j.pain.0000000000001229 29578943 PMC6053328

[B23] Hantman A. W. van den Pol A. N. Perl E. R. (2004). Morphological and physiological features of a set of spinal substantia gelatinosa neurons defined by green fluorescent protein expression. J. Neurosci. 24, 836–842. 10.1523/JNEUROSCI.4221-03.2004 14749428 PMC6729829

[B24] Haring M. Zeisel A. Hochgerner H. Rinwa P. Jakobsson J. E. T. Lonnerberg P.. (2018). Neuronal atlas of the dorsal horn defines its architecture and links sensory input to transcriptional cell types. Nat. Neurosci. 21, 869–880. 10.1038/s41593-018-0141-1 29686262

[B25] Harris K. D. Hochgerner H. Skene N. G. Magno L. Katona L. Bengtsson Gonzales C.. (2018). Classes and continua of hippocampal CA1 inhibitory neurons revealed by single-cell transcriptomics. PLoS Biol. 16:e2006387. 10.1371/journal.pbio.2006387 29912866 PMC6029811

[B26] Heinke B. Ruscheweyh R. Forsthuber L. Wunderbaldinger G. Sandkuhler J. (2004). Physiological, neurochemical and morphological properties of a subgroup of GABAergic spinal lamina II neurones identified by expression of green fluorescent protein in mice. J. Physiol. 560, 249–266. 10.1113/jphysiol.2004.070540 15284347 PMC1665197

[B27] Huang J. Polgar E. Solinski H. J. Mishra S. K. Tseng P. Y. Iwagaki N.. (2018). Circuit dissection of the role of somatostatin in itch and pain. Nat. Neurosci. 21, 707–716. 10.1038/s41593-018-0119-z 29556030 PMC5923877

[B28] Hughes D. I. Sikander S. Kinnon C. M. Boyle K. A. Watanabe M. Callister R. J.. (2012). Morphological, neurochemical and electrophysiological features of parvalbumin-expressing cells: a likely source of axo-axonic inputs in the mouse spinal dorsal horn. J. Physiol. 590, 3927–3951. 10.1113/jphysiol.2012.235655 22674718 PMC3476641

[B29] Jefferis G. S. Livet J. (2012). Sparse and combinatorial neuron labelling. Curr. Opin. Neurobiol. 22, 101–110. 10.1016/j.conb.2011.09.010 22030345

[B30] Koga K. Kanehisa K. Kohro Y. Shiratori-Hayashi M. Tozaki-Saitoh H. Inoue K.. (2017). Chemogenetic silencing of GABAergic dorsal horn interneurons induces morphineresistant spontaneous nocifensive behaviours. Sci. Rep. 7:4739. 10.1038/s41598-017-04972-3 28680103 PMC5498492

[B31] Konstantinides N. Kapuralin K. Fadil C. Barboza L. Satija R. Desplan C. (2018). Phenotypic convergence: distinct transcription factors regulate common terminal features. Cell 174, 622.e13–635.e13. 10.1016/j.cell.2018.05.021 29909983 PMC6082168

[B32] Kosugi M. Kato G. Lukashov S. Pendse G. Puskar Z. Kozsurek M.. (2013). Subpopulation-specific patterns of intrinsic connectivity in mouse superficial dorsal horn as revealed by laser scanning photostimulation. J. Physiol. 591, 1935–1949. 10.1113/jphysiol.2012.244210 23297304 PMC3624861

[B510] Larsson M. (2017). Pax2 is persistently expressed by GABAergic neurons throughout the adult rat dorsal horn. Neurosci. Lett. 638, 96–101. 10.1016/j.neulet.2016.12.015 27939388

[B33] Maxwell D. J. Belle M. D. Cheunsuang O. Stewart A. Morris R. (2007). Morphology of inhibitory and excitatory interneurons in superficial laminae of the rat dorsal horn. J. Physiol. 584, 521–533. 10.1113/jphysiol.2007.140996 17717012 PMC2277171

[B34] Mayhew J. A. Callister R. J. Walker F. R. Smith W. D. Graham B. A. (2019). Aging alters signaling properties in the mouse spinal dorsal horn. Mol. Pain 15:1744806919839860. 10.1177/1744806919839860 30845881 PMC6537084

[B35] Pagani M. Albisetti G. W. Sivakumar N. Wildner H. Santello M. Johannssen H. C.. (2019). How gastrin-releasing peptide opens the spinal gate for itch. Neuron 103, 102.e5–117.e5. 10.1016/j.neuron.2019.04.022 31103358 PMC6616317

[B36] Peirs C. Seal R. P. (2016). Neural circuits for pain: recent advances and current views. Science 354, 578–584. 10.1126/science.aaf8933 27811268 PMC11327866

[B37] Peirs C. Williams S. P. Zhao X. Walsh C. E. Gedeon J. Y. Cagle N. E.. (2015). Dorsal horn circuits for persistent mechanical pain. Neuron 87, 797–812. 10.1016/j.neuron.2015.07.029 26291162 PMC4562334

[B38] Petitjean H. Pawlowski S. A. Fraine S. L. Sharif B. Hamad D. Fatima T.. (2015). Dorsal horn parvalbumin neurons are gate-keepers of touch-evoked pain after nerve injury. Cell Rep. 13, 1246–1257. 10.1016/j.celrep.2015.09.080 26527000 PMC6038918

[B39] Polgár E. Durrieux C. Hughes D. I. Todd A. J. (2013). A quantitative study of inhibitory interneurons in laminae I-III of the mouse spinal dorsal horn. PLoS One 8:e78309. 10.1371/journal.pone.0078309 24205193 PMC3808353

[B40] Polgár E. Wright L. L. Todd A. J. (2010). A quantitative study of brainstem projections from lamina I neurons in the cervical and lumbar enlargement of the rat. Brain Res. 1308, 58–67. 10.1016/j.brainres.2009.10.041 19854164 PMC2828548

[B41] Punnakkal P. von Schoultz C. Haenraets K. Wildner H. Zeilhofer H. U. (2014). Morphological, biophysical and synaptic properties of glutamatergic neurons of the mouse spinal dorsal horn. J. Physiol. 592, 759–776. 10.1113/jphysiol.2013.264937 24324003 PMC3934713

[B42] Rall W. Burke R. E. Smith T. G. Nelson P. G. Frank K. (1967). Dendritic location of synapses and possible mechanisms for the monosynaptic EPSP in motoneurons. J. Neurophysiol. 30, 1169–1193. 10.1152/jn.1967.30.5.1169 4293410

[B43] Ross S. E. Hachisuka J. Todd A. J. (2014). “Spinal microcircuits and the regulation of itch,” in Itch: Mechanisms and Treatment, eds Carstens E. Akiyama T. (Boca Raton, FL: CRC Press/Taylor and Francis). 24830016

[B44] Sathyamurthy A. Johnson K. R. Matson K. J. E. Dobrott C. I. Li L. Ryba A. R.. (2018). Massively parallel single nucleus transcriptional profiling defines spinal cord neurons and their activity during behavior. Cell Rep. 22, 2216–2225. 10.1016/j.celrep.2018.02.003 29466745 PMC5849084

[B45] Schindelin J. Arganda-Carreras I. Frise E. Kaynig V. Longair M. Pietzsch T.. (2012). Fiji: an open-source platform for biological-image analysis. Nat. Methods 9, 676–682. 10.1038/nmeth.2019 22743772 PMC3855844

[B46] Smith K. M. Boyle K. A. Madden J. F. Dickinson S. A. Jobling P. Callister R. J.. (2015). Functional heterogeneity of calretinin-expressing neurons in the mouse superficial dorsal horn: implications for spinal pain processing. J. Physiol. 593, 4319–4339. 10.1113/jp270855 26136181 PMC4594251

[B47] Smith K. M. Boyle K. A. Mustapa M. Jobling P. Callister R. J. Hughes D. I.. (2016). Distinct forms of synaptic inhibition and neuromodulation regulate calretinin-positive neuron excitability in the spinal cord dorsal horn. Neuroscience 326, 10–21. 10.1016/j.neuroscience.2016.03.058 27045594 PMC4919388

[B48] Sun S. Xu Q. Guo C. Guan Y. Liu Q. Dong X. (2017). Leaky gate model: intensity-dependent coding of pain and itch in the spinal cord. Neuron 93, 840.e5–853.e5. 10.1016/j.neuron.2017.01.012 28231466 PMC5324710

[B50] Takazawa T. Choudhury P. Tong C. K. Conway C. M. Scherrer G. Flood P. D.. (2017). Inhibition mediated by glycinergic and GABAergic receptors on excitatory neurons in mouse superficial dorsal horn is location-specific but modified by inflammation. J. Neurosci. 37, 2336–2348. 10.1523/JNEUROSCI.2354-16.2017 28130358 PMC5354347

[B49] Takazawa T. MacDermott A. B. (2010). Synaptic pathways and inhibitory gates in the spinal cord dorsal horn. Ann. N Y Acad. Sci. 1198, 153–158. 10.1111/j.1749-6632.2010.05501.x 20536929 PMC2913540

[B51] Todd A. J. (2010). Neuronal circuitry for pain processing in the dorsal horn. Nat. Rev. Neurosci. 11, 823–836. 10.1038/nrn2947 21068766 PMC3277941

[B52] Torsney C. MacDermott A. B. (2006). Disinhibition opens the gate to pathological pain signaling in superficial neurokinin 1 receptor-expressing neurons in rat spinal cord. J. Neurosci. 26, 1833–1843. 10.1523/JNEUROSCI.4584-05.2006 16467532 PMC6793628

[B53] Walsh M. A. Graham B. A. Brichta A. M. Callister R. J. (2009). Evidence for a critical period in the development of excitability and potassium currents in mouse lumbar superficial dorsal horn neurons. J. Neurophysiol. 101, 1800–1812. 10.1152/jn.90755.2008 19176612

[B54] Wang L. Chen S. R. Ma H. Chen H. Hittelman W. N. Pan H. L. (2018). Regulating nociceptive transmission by VGluT2-expressing spinal dorsal horn neurons. J. Neurochem. 147, 526–540. 10.1111/jnc.14588 30203849 PMC6263733

[B55] Yasaka T. Tiong S. Y. Hughes D. I. Riddell J. S. Todd A. J. (2010). Populations of inhibitory and excitatory interneurons in lamina II of the adult rat spinal dorsal horn revealed by a combined electrophysiological and anatomical approach. Pain 151, 475–488. 10.1016/j.pain.2010.08.008 20817353 PMC3170912

[B56] Zeilhofer H. U. Studler B. Arabadzisz D. Schweizer C. Ahmadi S. Layh B.. (2005). Glycinergic neurons expressing enhanced green fluorescent protein in bacterial artificial chromosome transgenic mice. J. Comp. Neurol. 482, 123–141. 10.1002/cne.20349 15611994

[B57] Zeisel A. Muñoz-Manchado A. B. Codeluppi S. Lönnerberg P. La Manno G. Juréus A.. (2015). Brain structure. Cell types in the mouse cortex and hippocampus revealed by single-cell RNA-seq. Science 347, 1138–1142. 10.1126/science.aaa1934 25700174

[B58] Zeng H. Sanes J. R. (2017). Neuronal cell-type classification: challenges, opportunities and the path forward. Nat. Rev. Neurosci. 18, 530–546. 10.1038/nrn.2017.85 28775344

